# Preparation of Renewable Epoxy-Amine Resins With Tunable Thermo-Mechanical Properties, Wettability and Degradation Abilities From Lignocellulose- and Plant Oils-Derived Components

**DOI:** 10.3389/fchem.2019.00159

**Published:** 2019-03-27

**Authors:** Louis Hollande, Izia Do Marcolino, Patrick Balaguer, Sandra Domenek, Richard A. Gross, Florent Allais

**Affiliations:** ^1^URD ABI, CEBB, AgroParisTech, Pomacle, France; ^2^UMR GENIAL, AgroParisTech, INRA, Université Paris-Saclay, Massy, France; ^3^Institut de Recherche en Cancérologie de Montpellier, Val d'Aurelle, Montpellier, France; ^4^Department of Chemistry and Chemical Biology, Rensselaer Polytechnic Institute, Troy, NY, United States

**Keywords:** ferulic acid, glycerol, lipase, bio-based thermosets, wettability, degradability

## Abstract

One-hundred percent renewable triphenol—GTF—(glycerol trihydroferulate) and novel bisphenols—GDF_x_–(glycerol dihydroferulate) were prepared from lignocellulose-derived ferulic acid and vegetal oil components (fatty acids and glycerol) using highly selective lipase-catalyzed transesterifications. Estrogenic activity tests revealed no endocrine disruption for GDF_x_ bisphenols. Triethyl-benzyl-ammonium chloride (TEBAC) mediated glycidylation of all bis/triphenols, afforded innocuous bio-based epoxy precursors GDF_x_EPO and GTF-EPO. GDF_x_EPO were then cured with conventional and renewable diamines, and some of them in presence of GTF-EPO. Thermo-mechanical analyses (TGA, DSC, and DMA) and degradation studies in acidic aqueous solutions of the resulting epoxy-amine resins showed excellent thermal stabilities (*T*_d_5% = 282–310°C), glass transition temperatures (*T*_*g*_) ranging from 3 to 62°C, tunable tan α, and tunable degradability, respectively. It has been shown that the thermo-mechanical properties, wettability, and degradability of these epoxy-amine resins, can be finely tailored by judiciously selecting the diamine nature, the GTF-EPO content, and the fatty acid chain length.

## Introduction

Thermoset polymers are widely used in industrial applications thanks to their versatile performance, good durability, and excellent chemical resistance provided by their highly cross-linked structure (Ellis, 1993; Auvergne et al., 2014; Ramon et al., 2018). It is therefore very common to find these polymers in a broad range of applications such as maintenance coating, adhesives for aerospace (Prolongo et al., 2009) and automobile (Holbery and Houston, 2006) industries, or binders in composites (Gojny et al., 2006). Epoxy resins are one of the most important thermoset materials and are usually synthesized by reacting (poly)phenolic compounds with epichlorohydrin under basic conditions (Bruins, 1968).

The thermoset polymers sector, and more generally the polymer industry, has adopted the current trend which consists in developing greener and more sustainable chemicals and technologies. For instance, Solvay or Dow Chemical have already initiated the transition with the commercialization of bio-based epichlorohydrin prepared from the chlorination of bio-based glycerol, a by-product of biodiesel production (Strebelle et al., 2001; Freddy, 2006). Furthermore, a number of renewable resources—including fatty acids (Biermann et al., 2000; Maisonneuve et al., 2013; Laurichesse et al., 2014), lignocellulosic biomass (Isikgor and Becer, 2015), and enzymatic products—have been reported as alternative feedstocks in polymers synthesis.

Bisphenol A (BPA) is commonly used to prepare commercial high-performance epoxy resins (aka DGEBA, DiGlycidylEther of Bisphenol A). Although DGEBA, upon curing with diamines, leads to cross-linked materials with exceptional properties such as strong adhesion, mechanical integrity and chemical resistance, BPA is recognized as an endocrine disruptive chemical and many studies have been conducted to replace it by innocuous bio-based chemicals. Lignans and lignin-derived chemicals that can be obtained through pyrolysis (Celikbag et al., 2017; Barde et al., 2018) or controlled depolymerization (Pandey and Kim, 2011; Shuai et al., 2016) are the most used biomass-derived chemical platforms for the design of bio-based BPA-substitutes, for the preparation of sustainable thermosets with high thermo-mechanical properties. Indeed, the aromatic moieties present in these bio-based chemicals confer rigidity to the resulting polymers (Wang et al., 2017; Feghali et al., 2018). For example, there have been many reports on epoxy resins from eugenol (Wan et al., 2016), vanillin (Fache et al., 2015a,b; Hernandez et al., 2016; Mauck et al., 2017; Nicastro et al., 2018; Savonnet et al., 2018; Zhao et al., 2018), ferulic and sinapic acids (Maiorana et al., 2016; Janvier et al., 2017; Ménard et al., 2017), guaiacols (Maiorana et al., 2015), and creosol (Meylemans et al., 2011) with promising performances capable of competing with current DGEBA-based materials. However, few researchers have considered the potential toxicity of these BPA substitutes, especially endocrine disruption, which remains a crucial health issue for both plastic sector workers and consumers (Jiang et al., 2018). The degradability of the resulting materials and their behavior regarding water are two other aspects that are also under-investigated. In fact, in most of the studies described above, the thermo-mechanical (e.g., *T*_*g*_) and physico-chemical (e.g., wettability) properties of the materials cannot be finely tuned because of the lack of apolar moieties in the bio-based aromatic-epoxies.

Plant oil-derived fatty acids are highly valuable building blocks and are frequently used as renewable resources for the synthesis of thermoplastics/thermosets (Meier, 2018). Their long aliphatic chain and their double bond(s) allow a wide number of chemical modifications and provide materials with tunable properties, such as glass transition temperature (*T*_*g*_), chemical resistance, (bio)degradability, and adhesion strength. Combining the rigidity of bio-based aromatic building blocks and the apolar moiety of the alkyl chain of a fatty acid in a single BPA-substitute thus appears as an interesting approach to finely tune the *T*_*g*_, the wettability and the degradability of the resulting epoxy-amine resins, after curing with diamines.

In this present work, we aim at developing a series of bio-based epoxy resins, from naturally occurring ferulic acid and plant oils-derived components (i.e., glycerol and fatty acids), to access epoxy-amine thermoset materials with tunable thermo-mechanical properties, wettability and chemical degradation ability. Through highly selective chemo-enzymatic pathways, several polyphenolic compounds, named GDF_x_ and GTF, have been prepared with tunable flexibility and degree of functionality (i.e., 2 or 3). The determination of the toxicity of these compounds has been carried out by determining their binding to human hormone receptors (ERα, PXR, and AR) and was benchmarked against that of commercial and controversial BPA. GDF_x_ and GTF were then converted to their corresponding glycidyl ether by TEBAC-mediated glycidylation in presence of epichlorohydrin. Characterization studies were then performed on the corresponding epoxy resins formulations (i.e., molecular weight distribution and EEW). The latter were finally cured with three crosslinkers (i.e., IPDA, DA10, and DIFFA) and the thermo-mechanical properties as well as wettability and chemical degradation abilities of the resulting epoxy-amine resins were investigated.

## Experimental

### Material

Ferulic acid, lauric acid, palmitic acid, stearic acid, benzyl bromide, *N*,*N*-Dimethylpyridin-4-amine (DMAP), *N*,*N*-Diisopropylcarbodiimide (DIC), benzyltriethyl ammonium chloride (TEBAC), and palladium supported on carbon, were supplied by Sigma-Aldrich. Glycerol was purchased from Alfa Aesar. Epichlorohydrin was purchased from Acros Organics. Isophorone diamine (IPDA) and Decane diamine (DA10) were purchased from TCI. *Candida antarctica* Lipase B (CAL-B) immobilized on resin (LC200291, 10,000 propyl laurate units.g^−1^) was obtained from Novozyme. All reactants were used as received. All solvents were bought either from ThermoFisher Scientific or VWR. Deuterated chloroform (CDCl_3_) was purchased from Euriso-top.

### Methods

#### Purification

Column chromatographies were carried out with an automated flash chromatography (PuriFlash 4100, Interchim) and pre-packed INTERCHIM PF-30SI-HP (30 μm silica gel) columns using a gradient of cyclohexane and ethyl acetate for the elution.

#### Characterization

FT-IR analyses were performed on Cary 630 FT-IR with ATR. NMR analyses were recorded on a Bruker Fourier 300. ^1^H NMR spectra of samples were recorded in CDCl_3_ at 300 MHz, chemicals shifts were reported in parts per million (CDCl_3_ residual signal at δ = 7.26 ppm). ^13^C NMR spectra of samples were recorded at 75 MHz (CDCl_3_ signal at δ = 77.16 ppm). HRMS were recorded by the PLANET platform at URCA on a Micromass GC-TOF.

#### Gel Permeation Chromatography (GPC)

Gel permeation chromatography (GPC) was performed at 40°C on an Infinity 1260 system from Agilent Technologies with PL-gel 5 μM MIXED-D column in THF (flow rate 1 mL.min^−1^) using conventional PS calibration and UV detection at 280 nm.

#### Thermo-Gravimetric Analyses (TGA)

Thermo-gravimetric analyses (TGA) were recorded on a Q500, from TA. About 10 mg of each sample was heated at 10°C.min^−1^ from 30 to 500°C under nitrogen or oxygen flow (60 mL.min^−1^).

#### Differential Scanning Calorimetry (DSC)

Differential scanning calorimetry (DSC) thermograms were obtained using a DSC TA Q20, under inert atmosphere (N_2_), with a calibration using indium, *n*-octadecane and *n*-octane standards. For each sample, about 10 mg was weighed in a pan which was then sealed and submitted to three heat/cool/heat cycles: heating from 30 to 200°C at 10°C.min^−1^, cooling from 200°C to −50°C at 20°C.min^−1^. Glass transition temperatures (*T*_*g*_) were determined at the inflection value in the heat capacity jump.

#### Dynamical Mechanical Analyses (DMA)

Dynamical Mechanical Analyses (DMA) were performed on a DMA Q800 from the TA Instrument. The DMA samples had a rectangular geometry (length: 40 mm, width: 8 mm, thickness: 1.5 mm). Uniaxial stretching of samples was performed while heating at a rate of 3°C.min^−1^ from 30 to 200°C, keeping frequency at 1 Hz. Deformation was kept at 0.001% (amplitude of 7 μm) to stay in the linear viscoelastic region. The storage modulus (E') and tan δ curves as a function of the temperature, were recorded and analyzed using the TA Instruments Universal Analysis 2000 software. The temperatures *T*_α_ *(*°*C)* were determined as the temperatures at the peak maximum of the tan δ curves.

#### Synthesis of Resin Epoxy Precursors

**Scheme 1:**
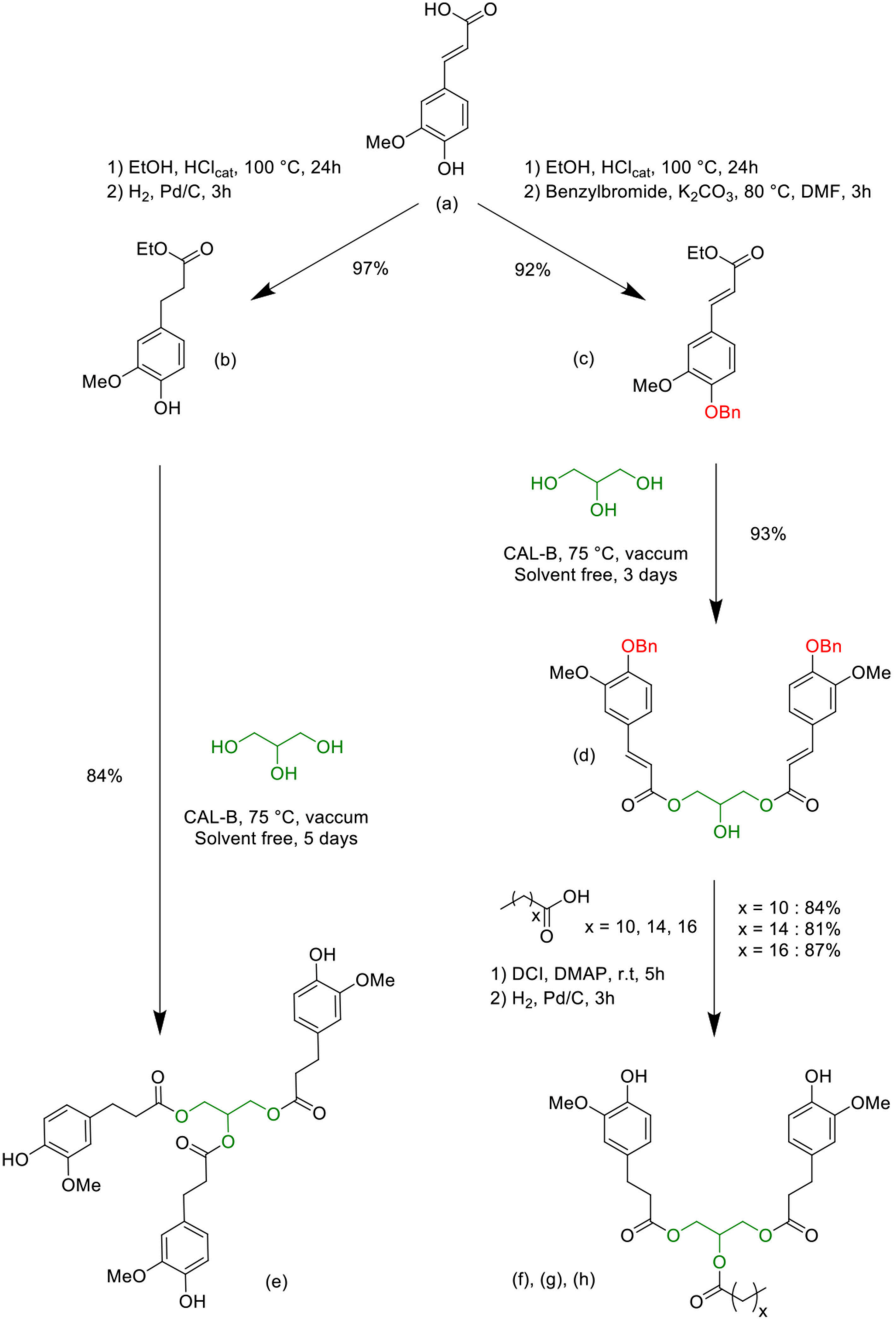
Syntheses of the bio-based bisphenols (GDF_x_) (f), (g), (h), and triphenol (GTF) (e) precursors from ferulic acid, glycerol and fatty acids.

Scheme 1 presents the complete synthetic pathway using the following molecules: ferulic acid (**a**), ethyl dihydroferulate (**b**), benzylated ethyl ferulate (**c**), benzylated glycerol diferulate (GDFoBn) (**d**), glycerol tri-dihydroferulate (GTF) (**e**) and lipophilic glycerol dihydroferulates (GDF_x_) (**f**, **g** and **h**). Compound syntheses were carried out following procedures previously described in the literature (Ménard et al., 2017; Hollande and Domenek, 2018). Full characterizations and detailed procedures for new compounds are given in the Electronic Supplementary Information (ESI). ^1^H & ^13^C NMR spectra, FTIR spectrum as well as TGA trace of GTF-EPO can be found in a previous published work (Ménard et al., 2017).

#### Glycidylation

Phenolic precursors (GDF_x_ or GTF; 1 eq) were dissolved in epichlorohydrin (10 eq/OH). Triethyl benzyl chloride (TEBAC) (0.2 eq) was added and the suspension was stirred for 2 h at 80°C. The reaction medium was cooled down to room temperature and NaOH (5M, 2 eq/OH) was added. The biphasic solution was vigorously stirred for 4 h at room temperature then extracted with ethyl acetate (3 × 100 mL). The organic layers were washed with brine (80 mL), dried over anhydrous MgSO_4_, filtered and concentrated *in vacuo*.

#### Epoxide Equivalent Weight (EEW)

Experimental values of EEW for the epoxide resins synthesized herein were determined by ASTM D1652. Samples were dissolved in dichloromethane (DCM) with tetraethylammonium bromide and titrated in triplicate, using perchloric acid solution in acetic acid (1 N). Crystal violet was used as an indicator and the end point was determined when the solution turned from blue to green for longer than 30 s. Detailed results are provided in the ESI.

#### Preparation of Cured Epoxy Materials

This method considers that the epoxy resins prepared herein contained small amounts of higher molecular weights fractions. The weight of the resins and cross-linkers were determined by using the EEW values and amine hydrogen equivalent weight (AHEW). Weights of reaction components for the formulated resin systems were determined using equation (1). Equations (2) and (3) were used to calculate the AHEW and to obtain the parts by weight of diamine per hundred parts resin (phr).

(1) $$\begin{array}{r} EEW\;of\;mix=\;\frac{Total\;Weight}{\frac{Weight\;of\;resin\;A}{EEW\;of\;resin\;A}+\;\frac{Weight\;of\;resin\;B}{EEW\;of\;resin\;B}} \end{array}$$

(2) $$\begin{array}{r} AHEW=\;\frac{molecular\;weight\;of\;amine}{number\;of\;active\;hydrogens} \end{array}$$

(3) $$\begin{array}{r} phr=\;\frac{AHEW\;x\;100}{EEW} \end{array}$$

For instance, if phr = 20, then 20 g of diamine would be needed for 100 g of epoxy resin. Epoxy resins were vigorously mixed with the appropriate amount of crosslinker at room temperature in a disposable aluminum pan. In the case of the solid diamine DA10, an additional stirring time at 120°C was necessary to obtain a complete dissolution of the amine in the epoxy precursor. Mixtures were then transferred into rectangular stainless steel molds (length: 40 mm, width: 8 mm, thickness: 1.5 mm). The method of curing was adapted from a literature procedure (Janvier et al., 2017). In summary, the resins were maintained under ambient conditions in the molds for 2 h and then for 2 h at 60°C, for 20 h at 110°C and for 1 h at 150°C.

#### Hydrolytic Degradation Assays

Cured resin samples with dimensions about 4 × 8 × 1.5 mm, with weights ranging from 75 to 95 mg, were placed in a 10 mL sealed tube. An aqueous solution of HCl (3M) was added. The vials were heated at 60°C for 40 min for equilibration. Incubations were then continued for 5, 20, 26, 43, and 50 h. The residual solid was then washed with deionized water, dried then weighed to determine the mass loss.

#### Measurement of Contact Angle

The contact angle (θ) of liquid water against the cured epoxy resins was measured by the sessile drop method at room temperature with a drop shape analysis system (DIGIDROP and Visiodrop software). The contact angle was measured at three different locations for each thermoset and the average value was reported.

#### Estrogen Binding Affinity Testing

The agonistic and antagonistic potentials of bisphenols were analyzed following a literature method (Delfosse et al., 2012) in which ERα, PXR, and AR transcriptional activities were monitored by using corresponding reporter cells HELN ERα, U2OS AR, and HG5LN PXR cells, respectively. Activities were measured in relative light units (RLU) and 100% activities were assigned to the RLU value obtained with 10 nM agonist control (estradiol (E_2_), R 1881, SR 12813). The vehicle (DMSO) was tested as a control without any compound.

## Results and Discussion

### Synthesis of the Bio-Based Epoxy Resin

#### Chemo-Enzymatic Synthesis

Chemical esterification of *p-*hydroxycinnamic acids with alcohol is unselective and side reactions generally lead to an unwanted product that needs to be removed by purification steps, generating waste to dispose of. In opposition, the lipase-mediated enzymatic synthesis offers some advantages for synthesizing esters, such as milder reaction conditions and selectivity. Hence, the selective synthesis of the bis/tri-ferulate resin epoxy precursors **e**, **f**, **g** and **h**, involving glycerol linker group, was based on chemo-enzymatic methods recently published (Scheme 1) (Pion et al., 2013; Hollande and Domenek, 2018).

Ferulic acid was first transformed either in ethyl dihydroferulate (Scheme 1, b) or in benzylated ethyl ferulate (Scheme 1, c), through a two-step one-pot process. Afterwards, a specific affinity of *Candida antarctica* Lipase B (CAL-B) toward glycerol allowed controlling the degree of functionalization of the latter, giving access to the symmetric bisphenol (Scheme 1, d) or to the fully functionalized glycerol triferulate (Scheme 1, e) in excellent yields and purities. Fatty acids were then grafted onto the available secondary alcohol of the intermediary compound d, immediately followed by a palladium-catalyzed hydrogenation to simultaneously reduce unsaturation and cleave the benzyl group, thus providing the GDF_x_ compounds (Scheme 1, f, g, h. It is noteworthy to mention that all the syntheses described in Scheme 1 have been performed at the multi-gram scale. Moreover, in a recent work, we have not only demonstrated the feasibility of the lipase-mediated transesterification of dihydroferulate ethyl ester at the kilo-scale (Teixeira et al., 2017), but also devised a sustainable and industrially relevant membrane-based technique, allowing both the purification of the targets and the recycling of solvents and unreacted reagents.

#### Endocrine Receptor Activities of GDF_x_ Bisphenols

**Figure 1:**
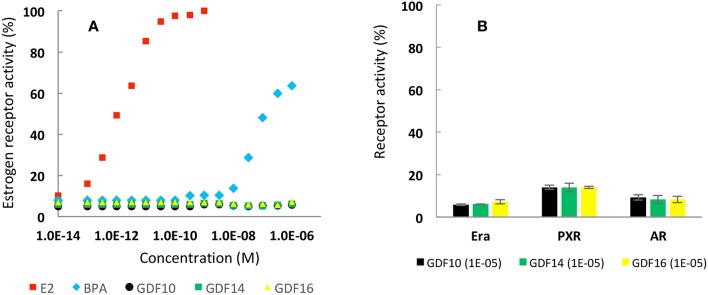
**(A)** Estrogenic activity (%) as function of concentration of E2, BPA and synthesized bisphenols. **(B)** Activities (%) of Erα, PXR and AR induced by synthesized bisphenol at 10^−5^ molarity.

Nowadays, the development of new platform chemicals is deeply regulated, especially for bisphenolic compounds—such as BPA—and other controversial endocrine disrupting chemicals (EDCs). Herein, endocrine activities of newly created GDF_x_ bisphenols were investigated by evaluating their ability to interact with three types of receptors ERα, PXR, and AR. ERα is a member of the nuclear hormone receptors family, and its activity is regulated by the steroid estrogen sex hormone 17β-estradiol (E2). PXR is a member of the steroid and xenobiotic sensing nuclear receptors family and AR is a nuclear type androgen receptor that is activated by binding with any of the androgenic hormones. In Figure 1A, sex hormone E2 induces a 100% ERα activity at 5.10^−10^ M, which plateaus at higher concentrations. For BPA, estrogenic activity was observed at molarities of 10^−7^ and slightly above 60% at concentrations of 10^−5^. In contrast, all newly created GDF_x_ bisphenols, GDF_10_, GDF_14_, and GDF_16_, exhibited an activity similar to that of the control, even with increasing concentrations. In addition, Figure 1B reveals that the highest concentration tested for GDF_x_ family (i.e., 10^−5^ M) did not induce any abnormal receptor activities on both PXR and AR. Finally, an endocrine disruption assay (ESI p22) indicated that phenolic structures were neither agonist nor antagonist ligands. Based on these results, that demonstrated the innocuousness of these newly created GDF_x_ bisphenols, we were encouraged to further explore their use for the production of epoxies and epoxy-amine resins.

#### Glycidylation

**Scheme 2:**
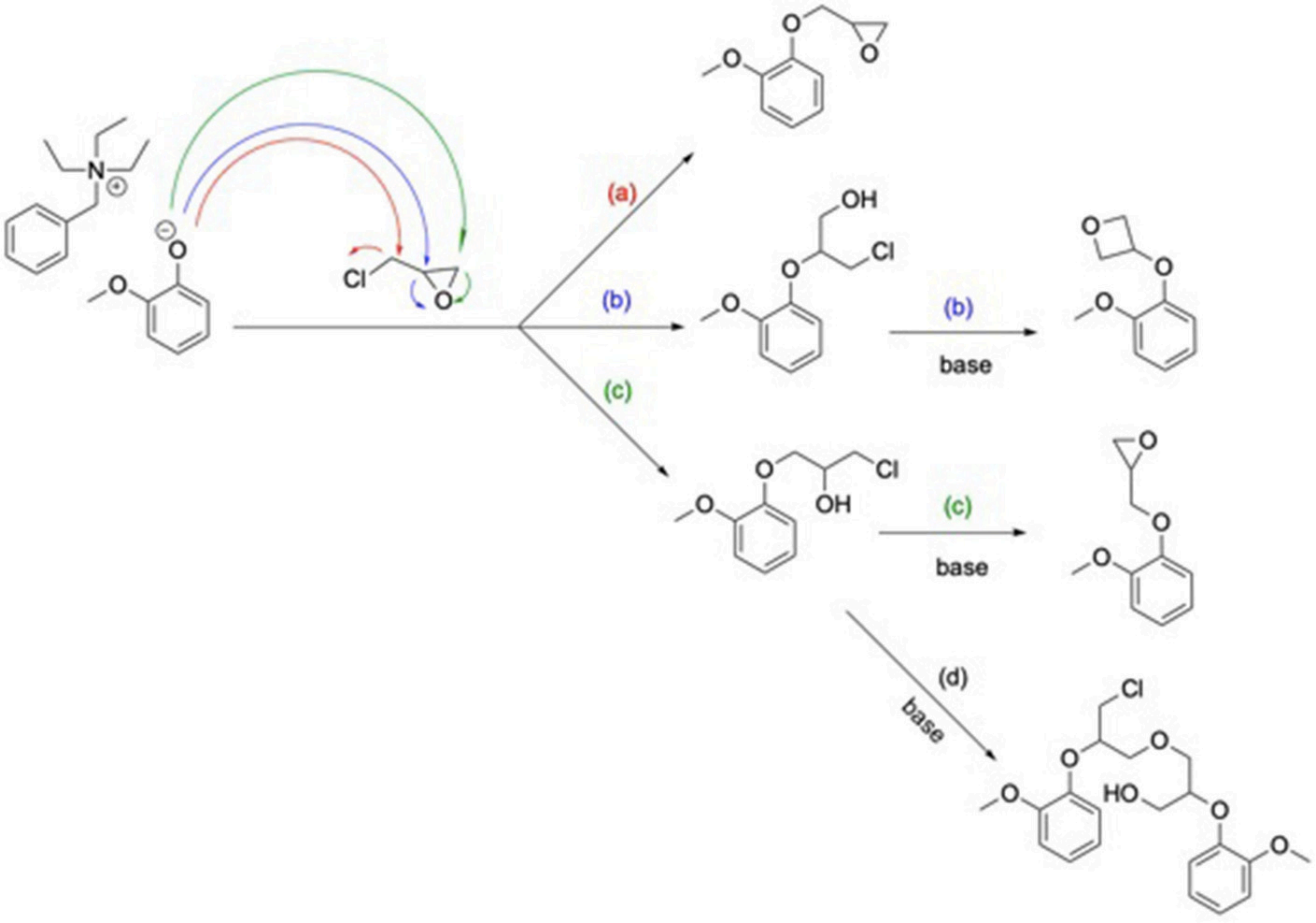
Glycidylation mechanism by SN_2_ (a) or epoxy ring opening (c) and oxetane (b) or oligomeric product (d) formation consequent to a basic treatment.

Epoxy resins synthesis consists in the glycidylation of the different GDF_x_ bisphenols, using the classical procedure, i.e., large excess of epichlorohydrin under alkaline conditions. However, to prevent the partial hydrolysis of the internal ester moieties observed by Maiorana et al. (2016) during classical glycidylation of bisferulate esters, milder temperatures, and shorter reaction times were applied. Thus, to offset this restriction, a catalytic amount of triethylbenzyl ammonium chloride (TEBAC) as phase transfer catalyst—well known for allowing higher epoxy functionality—was used (Fache et al., 2015a). Under such conditions, the phenolate formed is able to attack each one of the carbons of epichlorohydrin (Scheme 2), two of them, (b) and (c), leading to the epoxy ring opening. Thus, a basic treatment is necessary to close the open forms. Yet, not all the resulting species are diepoxies resins. Side reactions can occur, the most important being (b) the oxetane formation or (d) the formation of branched molecules due to the oligomerization of chlorinated intermediate (Ellis, 1993).

**Figure 2:**
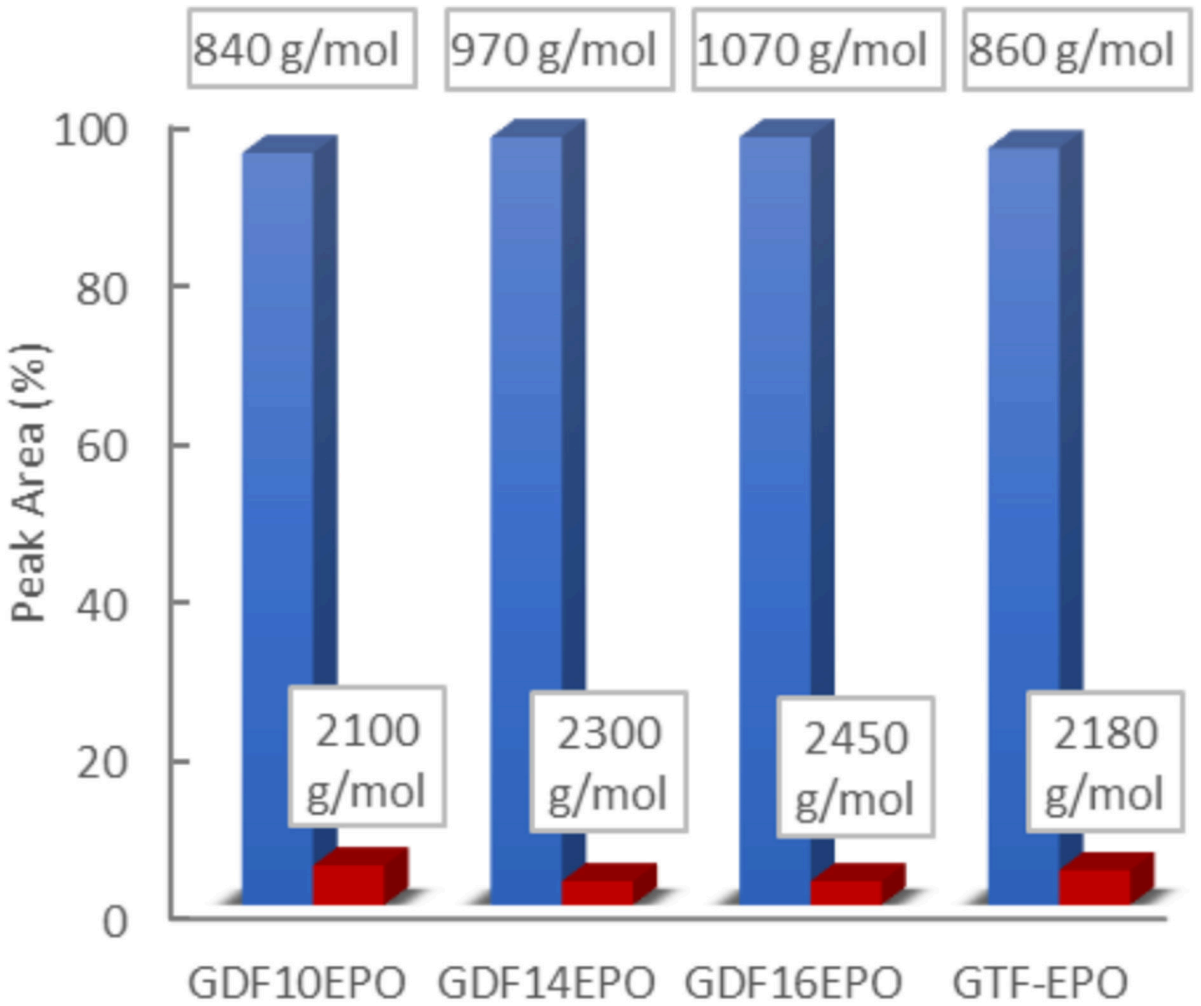
Relative molecular weight distribution (%) of epoxy resins determined by GPC.

Resulting crude di/triglycidyl ether bis/triferulate epoxy resins, named GDF_x_EPO, and GTF-EPO, respectively, were obtained in high isolated yields (>85%). All the resins prepared herein (see ESI, p18) showed the appearance of an epoxy vibrational band at 912 cm^−1^ characteristic of oxirane C-O group on their FT-IR spectra. ^1^H NMR analyses showed the expected chemical shifts for the proton on the tertiary carbon of the oxirane ring at 3.40 ppm (see ESI, p17). However, signals were also observed between 3.75 and 3.95 ppm, which could be attributed to the oxetane or the oligomeric production epoxies pictured in Scheme 2 (b) and (d). In order to investigate the potential side formation of such oligomeric epoxies, molecular weight distributions of GDF_x_EPO and GTF-EPO resins were measured by gel permeation chromatography. The results are shown in Figure 2 and the ESI (p20-21).

The distribution of molecular species (*M*_*n*_) in epoxy resins was analogous for each sample. Experimentally determined values (GPC) described the normalized weight distribution of epoxy systems in two monodisperse fractions according to the *M*_*w*_/*M*_*n*_ values closed to 1.0. The first corresponds to the monomeric glycidyl ether of GDF_10_, GDF_14_, GDF_16_, and GTF, with *M*_*n*_ *of* 840, 970, 1,070, and 860 g/mol, respectively, whereas the second corresponds to *M*_*n*_ above 2,000 g/mol, suggesting the formation of oligomeric by-products through the dimerization of chlorinated intermediate depicted in Scheme 2. Nevertheless, the contribution of the high molecular weight tail to the cumulative molecular weight distribution is <5%. Considering the relatively low concentration of by-products estimated through ^1^H NRM and GPC analyses, we decided to use these epoxy resins directly without further purification. Table 1 displays values of experimental and theoretical epoxide equivalent weights (EEW) where the latter assumes 100% conversion to the corresponding monomeric glycidyl ferulate structures. Experimental EEWs values are 11 to 15% greater than that of the corresponding theoretical values. This result is attributed to the formation of dimers which results in the increase of the EEW of the synthesized resins above the theoretical value. Finally, Table 1 also reports on the thermal behaviors of uncured resins. TGA analysis revealed thermostability (T_d_ 5%) in the range of 311–340°C under an inert atmosphere, and 304–321°C under an oxidative atmosphere. Furthermore, the alkyl chain length does not significantly impact the degradation temperature.

**Table 1:** Epoxy resins characterization.

**Resin type**	**Epoxy content**			Thermal analysis^*^		
	**EEW_**exp**_**	**EEW_**theore**_**	**Increase (%)**	**T_d_ 5% (°C)^a^**	**T_d_ max (°C)^a^**	**T_**o**_ 5% (°C)^b^**
GDF_10_EPO	426 ± 6	371	13	317	369	314
GDF_14_EPO	471 ± 5	400	15	311	396	304
GDF_16_EPO	463 ± 9	414	11	340	371	321
GTF-EPO	303 ± 5	265	13	x	x	x

* *Determined by TGA (10°C.min^−1^)*.

a *Under N_2_ flow*.

b *Under O_2_ flow*.

### Thermosets Synthesis

**Figure 3:**
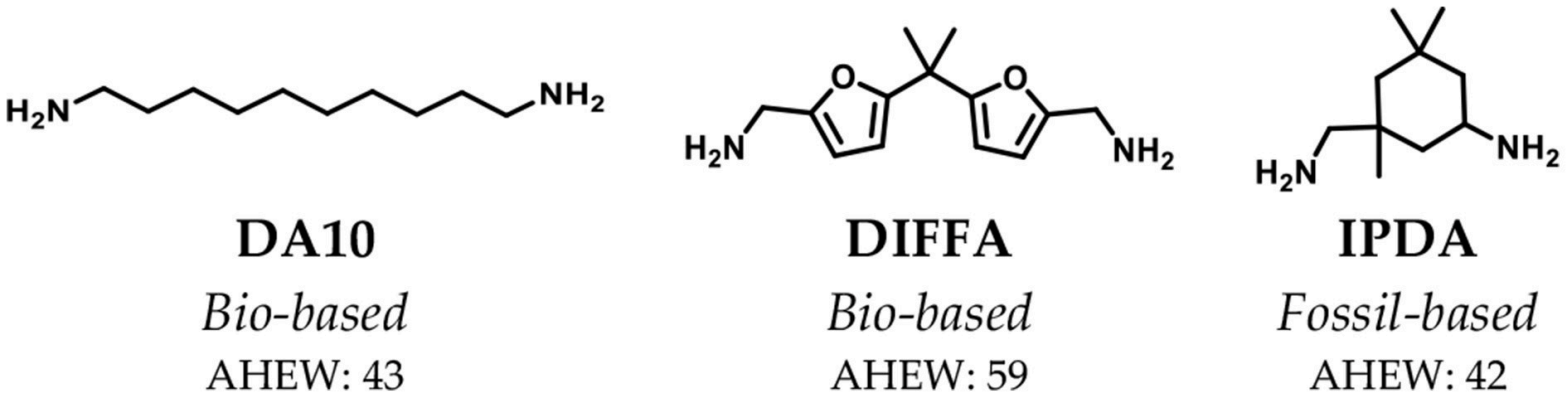
Chemical structures of the curing agents used for thermoset formulation.

The chemical structures of all amine hardeners or curing agents are reported in Figure 3. Two bio-based (DA10 and DIFFA) and one extensively used fossil-based (IPDA), presenting different rigidities, were selected.

**Figure 4:**
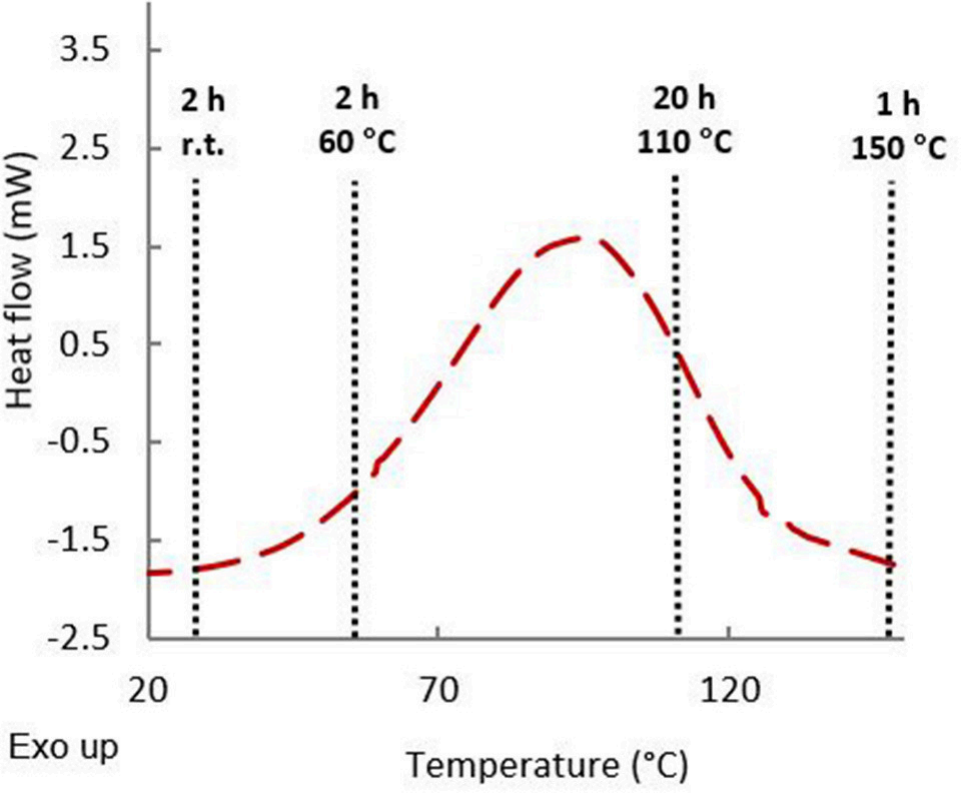
DSC analysis of an equimolar mixture of GDF_10_EPO and curing agent (IPDA).

To ensure that epoxy resin has a good reactivity toward diamines, a DSC analysis of an equimolar mixture of resins and diamines was carried out (Figure 4). An exothermic peak was observed around 100°C corresponding to the epoxy ring-opening reaction to form the crosslinked polymer. In order to ensure chain mobility during gelation and to obtain optimal crosslinking content, a multistep temperature program was elaborated. Mixtures were first left for 2 h at room temperature followed by two additional hours at 60°C. Formulations were then cured at T ≈ T_DSCcrosslinkingpeak_ (20 h at 110°C), then at T > T_DSCcrosslinkingpeak_ (1 h at 150°C). Each difunctionalized GDF_x_EPO epoxy precursor was cured in presence of each curing agent leading to 9 formulations (Table 2, thermosets 1–9). In order to tune the properties of these thermosets, and as the formulations with diamines and difunctionalized GDF_x_EPO (i.e., thermosets 1–9) did not provide materials rigid enough to perform DMA analyses, 1 formulation with GTF-EPO (Table 2, thermoset 15) as well as 5 co-formulations with IPDA, in variable ratios of difunctionalized (GDF_14_EPO) and tri-functionalized (GTF-EPO) epoxy resin (Table 2, thermosets 10–14) were also prepared.

**Table 2:** Results of the analyses of thermosets (ATG, DSC, DMA).

**Thermoset**	**Curing agent**	**Resin type**	***T_***d***_5%* (°C)^a^**	***Td_***max***_* (°C)^a^**	**_**W**_% char^a^**	***T_***g***_*(°C)^b^**	***T_**α**_* (°C)^c^**	**E^**′**^_**glassy**_ (Mpa)^c^**	**E^**′**^_**elastic**_ (Mpa)^c^**
1	DA10	GDF_10_EPO	296	356	9.9	5	x	x	X
2	DA10	GDF_14_EPO	299	356	7.7	4	x	x	x
3	DA10	GDF_16_EPO	306	365	8.6	3	x	x	x
4	DIFFA	GDF_10_EPO	282	368	16.9	15	x	x	x
5	DIFFA	GDF_14_EPO	286	378	13.6	11	x	x	x
6	DIFFA	GDF_16_EPO	282	376	15.9	16	x	x	x
7	IPDA	GDF_10_EPO	310	369	9.0	21	x	x	x
8	IPDA	GDF_14_EPO	298	352	10.9	23	x	x	x
9	IPDA	GDF_16_EPO	297	367	10.0	18	x	x	x
10	IPDA	87%-GDF_14_EPO 13%-GTF-EPO	290	378	10.1	25	x	x	x
11	IPDA	75%-GDF_14_EPO 25%-GTF-EPO	287	373	9.7	28	x	x	x
12	IPDA	50%-GDF_14_EPO 50%-GTF-EPO	289	371	14.2	45	61	678	3.5
13	IPDA	25%-GDF_14_EPO 75%-GTF-EPO	282	372	15.9	54	63	1,985	7.5
14	IPDA	10%-GDF_14_EPO 90%-GTF-EPO	266	371	18.7	60	71	2,523	9.7
15	IPDA	GFT-EPO	279	372	17.5	62	72	1,685	8.8

a *Determined by TGA (10°C.min^-1^, N_2_ flow)*.

b *Determined by DSC on the second heat-cool-heat cycle (10°C.min^-1^, N_2_ flow)*.

c *Determined by DMA (frequency 1 Hz, amplitude 7 μm, 3°C.min^-1^). E'_glassy_ at 30°C, E'_elastic_ at 150°C*.

### Characterization of the Thermosets

#### Thermogravimetric Analyses (TGA)

The thermal stability of cured thermosets was studied by thermal gravimetric analysis (TGA) under nitrogen flow. *T*_*d*_*5%* was defined as the temperature at which the thermosets lost 5% of its initial mass, *Td*_*max*_ as the temperature at which the kinetic degradation of the thermoset occurs at the maximum rate and W%_char_ corresponds to the relative amount of stable residue at a high temperature. Table 2 sums up the values for the thermosets prepared and all thermograms are displayed in ESI p23-25. The lowest *T*_*d*_*5%* was obtained with DIFFA (Table 2; thermosets 4–6), which might be explained by the initiation of the degradation by rupture of the curing agent segment, as reported by Ménard et al. (2017). Overall, higher *T*_*d*_*5%* and *Td*_*max*_were measured with IPDA- and DA10-containing resins. Concerning the high temperature stable char content (W%_char_), values were around 10% for GDF_x_EPO resins; moreover, the higher the GTF-EPO ratio in epoxy resin system, the higher the char content. This parameter would be particularly interesting for the development of flame-retardant materials.

#### Differential Scanning Calorimetric Analyses (DSC)

**Figure 5:**
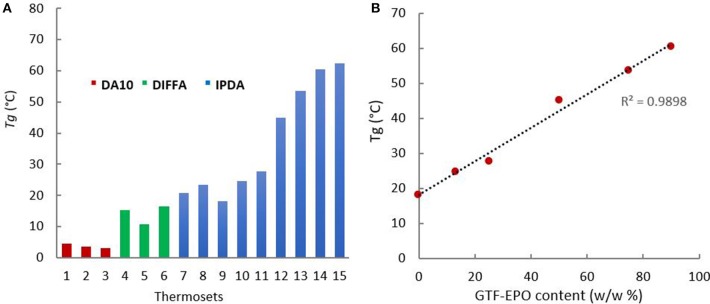
**(A)** *T*_g_ for thermosets formulated with (red) DA10, (green) DIFFA, and (blue) IPDA; **(B)** *Tg* as a function of GTF-EPO resin content.

The degree of cure of the resins and glass transition temperatures were both determined through DSC. Analyses of post-cured resins showed no residual exothermic peak above or below the glass transition corresponding to further curing reactions (ESI, p26-28). These results indicate that if additional cure ever occurred during DSC heating, it is below the instrument level of detection. Therefore, consumption of epoxy moieties and concurrent events of cross-linking during the curing cycle adopted herein results in a degree of cure that closely approaches its upper limit. Furthermore, the curing of difunctionalized resins (Table 2, entries 1–9 and 15) with hardeners presenting various rigidities (DA10 < DIFFA < IPDA due to their aliphatic, aromatic, and cyclic configuration), respectively, lead to a restricted range of *T*_*g*_ values (from 3 to 23°C). Hence, the most flexible amine (DA10) provided the lowest *T*_*g*_ values, while the IPDA-containing thermosets led to the highest *T*_*g*_. Nevertheless, for a given diamine, thermosets exhibit quite similar *T*_*g*_, meaning that the impact of the diamine on the rigidity of the network outweighs that of the alkyl chain length (Figure 5A), Entries 1–3; 4–6; 7–9).

When IPDA reacted with tri-functionalized resins, i.e., GTF-EPO, the *T*_*g*_ value increased up to 60°C due to the higher epoxy functionally (2 vs. 3) and higher cross-link density (Figure 5A, 15). Moreover, as shown in Figure 5B, it is noteworthy to mention that the *T*_*g*_ increased linearly with the tri-functionalized resin GTF-EPO content (*R*^2^ = 0.9898).

#### Dynamic Mechanical Analyses (DMA)

**Figure 6:**
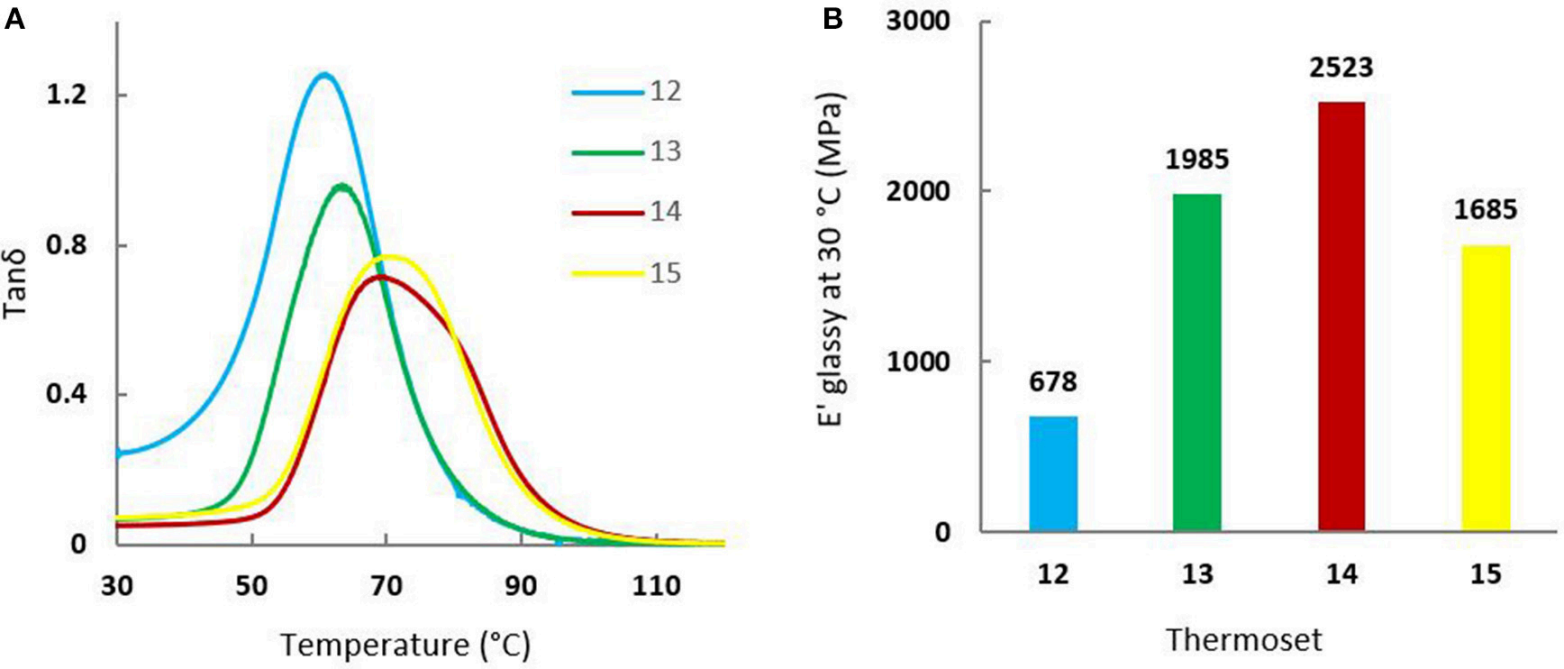
**(A)** Tan δ curves of (blue) 12, (green) 13, (red) 14, and (yellow) 15 thermosets; **(B)** Storage modulus (E'_glassy_) at 30°C of (blue) 12, (green) 13, (red) 14, and (yellow) 15 thermosets.

Mechanical properties of the cured resins were evaluated by DMA on each sample with a glass transition upwards of 30°C (thermoset 12–15). Figure 6 and Table 2 provide (a) tan δ values as a function of temperature and (b) storage modulus values at the glassy state at 30°C. Similar to the previous linear relationship observed for *T*_*g*_ and GTF-EPO_wt%_, the same trend was observed for tan δ that increased with the increasing incorporation of the GTF-EPO to the GDF_14_EPO system. As discussed previously, the thermal behaviors observed with the addition of GTF-EPO was a consequence of the decrease of EEWs of the co-formulated systems relative to the GDF_x_EPO resin. In other words, co-formulated resins will lead to thermosets with relatively higher crosslink densities. On the other hand, when the ratio of GDF_14_EPO in GTF-EPO does not exceed 25 wt%, thermosets exhibit a higher storage modulus than that of the GTF-EPO resin (Figure 6B; entries 13 and 14 vs. 15). It is worth mentioning that the storage modulus at 30°C of epoxy systems which contain 10 wt% of GDF_14_EPO is 50% higher than that of the GTF-EPO thermoset (2,523 vs. 1,685 MPa). This rather unexpected outcome may be explained by the more efficient interaction/distribution of lipophilic GTFxEPO epoxies during the gelation/curing induced by their “surfactant” properties.

#### Degradation and Wettability Behavior

To gain further insight on how surface hydrophobicity varies as a function of the alkyl chain length, contact angles were measured (Table 3). Whatever the curing agent, increasing the alkyl chain length from 12 to 18 carbons (i.e., lauric acid -> palmitic acid -> stearic acid) resulted in an increase of the contact angle (Table 3; thermosets 1 vs. 3, 4 vs. 6, and 7 vs. 9). However, it is worth mentioning that the nature of the curing agent does not significantly impact the hydrophilicity of thermosets as for a same resin GDF_x_EPO cured with the three different curing agents (i.e., DA10, DIFFA, and IPDA) contact angles differ by < ±7°.

**Table 3:** Characterization of weight loss plots and contact angle.

**Thermoset**	**Resin type**	**Curing agent**	**Weight loss/hour 10^**−3**^ (mg.h^**−1**^)**	**Least squares fitting value for weight loss plots (*R*^2^)**	**Contact angle (°)**
1	GDF_10_EPO	DA10	−17.1	0.965	66 ± 2
2	GDF_14_EPO	DA10	−15.8	0.997	75 ± 5
3	GDF_16_EPO	DA10	−2.2	0.990	82 ± 6
4	GDF_10_EPO	DIFFA	−17.3	0.997	63 ± 8
5	GDF_14_EPO	DIFFA	−12.8	0.983	76 ± 6
6	GDF_16_EPO	DIFFA	−5.2	0.939	83 ± 5
7	GDF_10_EPO	IPDA	−20.1	0.981	79 ± 3
8	GDF_14_EPO	IPDA	−14.7	0.854	82 ± 1
9	GDF_16_EPO	IPDA	−10.6	0.947	88 ± 4

**Figure 7:**
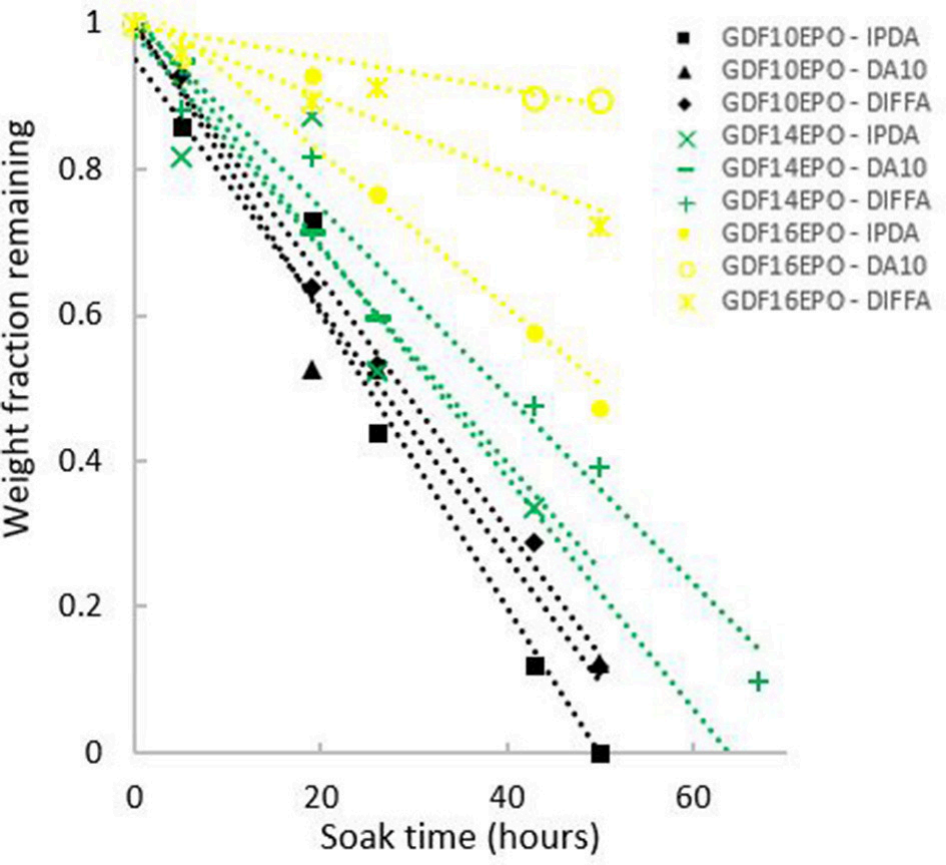
Analyses of thermoset weight loss as function of incubation time at 60°C in acid aqueous solutions.

In addition, thermosets weight loss, determined as a function of the incubation time in acidic aqueous solutions, was plotted (Figure 7). Data showed a decrease in mass upon incubation with linear behavior, according to the *R*^2^ values for weight loss plots (Table 3). Finally, it is noteworthy to mention that the length of the fatty acid chain grafted onto GDF_x_ epoxies had a significant effect on the hydrolytic degradation rate of cured thermosets. Indeed, an increase in hydrophobicity of the thermoset, by increasing the length of the fatty acids graft, was consistent with a corresponding decrease of the degradation rate measured in mg.h^−1^ and the susceptibility of that surface to hydrolysis.

## Conclusion

Herein, the chemo-enzymatic synthesis, thermo-mechanical properties, wettability and acidic hydrolysis of 100% renewable ferulic-, glycerol- and fatty acids-based bis- and triphenols - GDF_x_ and GTF - are described. The estrogenic activity of GDF_x_ with fatty acid chain lengths of 12, 16, and 18-carbons was quantified and compared to bisphenol A and 17β-estradiol and showed no significant activity for ERα, PXR, and AR receptors. Bio-based GDF_x_ and GTF bis/triphenols were then successfully converted to their corresponding di- and triglycidyl ether epoxy resins (i.e., GDF_x_EPO and GTF-EPO, respectively) through a TEBAC-mediated glycidylation. GDF_x_EPO was then cured with DIFFA, DA10 and IPDA diamines; GDF_14_EPO was also co-formulated with tri-functionalized GTF-EPO and IPDA. All the resulting epoxy-amines exhibited relatively high thermostability with *T*_*d*_*5%* ranging from 282 to 310°C and *T*_*g*_ between 3 and 62°C. Surprisingly, in the case of GDF_x_EPO-diamine resins, for a given diamine, the chain length of the fatty acid moiety did not significantly impact the *T*_*g*_. However, one can tailor the *T*_*g*_ by playing with the diamine nature and achieve *T*_*g*_ up to 23°C with IPDA and GDF_14_EPO. To further tailor the *T*_*g*_ and to reach higher values, GTF-EPO must be added to the formulation. In such formulations, DSC and DMA analyses showed that the *T*_*g*_ and the storage modulus can also be modulated by finely adjusting the GTF-EPO content. Finally, with regards to wettability and degradability, the chain length of the fatty acid was found to provide a simple but powerful approach to tailor the wettability and the susceptibility to hydrolysis of the GDF_x_EPO-based epoxy-amine resins. Indeed, the shortest fatty acid provided the highest wetting and hydrolysis rate, and *vice-versa*. This study therefore demonstrates the great potential of combining ferulic acid, glycerol and fatty acids using chemo-enzymatic processes for the preparation of epoxies and epoxy-amine networks with tunable properties.

## Author Contributions

FA and RG conceived the research. FA and SD managed the research. LH and ID performed the chemo-enzymatic reactions and the characterizations. PB performed the endocrine disruption assays. FA and LH provided the technical guidelines, reviewed the results, wrote, and drafted the article. FA, RG, and SD reviewed and approved the article.

### Conflict of Interest Statement

The authors declare that the research was conducted in the absence of any commercial or financial relationships that could be construed as a potential conflict of interest.
