# Supplementary material for: Preparation of Renewable Epoxy-Amine Resins With Tunable Thermo-Mechanical Properties, Wettability and Degradation Abilities From Lignocellulose- and Plant Oils-Derived Components
Source: Front Chem. 2019 Mar 27;7:159. doi: 10.3389/fchem.2019.00159 (PMC6445855; doi:10.3389/fchem.2019.00159)

# Preparation of renewable epoxy-amine resins with tunable thermo-mechanical properties, wettability and degradation abilities from lignocellulose- and plant oils-derived components

Hollande,^a,b^ L.; Do Marcolino,^a^ I.; Balaguer,^d^ P.; Domenek,^b^ S.; Gross,^c^ R. ; Allais,*^a^ F.

^a^ Chaire ABI, AgroParisTech, CEBB, 3 rue des Rouges Terres 51110 Pomacle, France

^b^ UMR GENIAL, AgroParisTech, INRA, Université Paris-Saclay, Avenue des Olympiades, 91300 Massy, France

^c^ Department of Chemistry and Chemical Biology, Rensselaer Polytechnic Institute, Troy, New York 12180, United States

^d^ Institut de Recherche en Cancérologie de Montpellier, Campus Val d’Aurelle, 208 rue des Apothicaires, 34298 Montpellier Cedex 5, France

[florent.allais@agroparistech.fr](mailto:florent.allais@agroparistech.fr)

Electronic Supplementary Information

Table of contents

1

Monomers 4

Published synthetic pathways 4

^1^H NMR spectrum of benzylated ethyl ferulate (CDCl_3_) 6

^1^H NMR spectrum of GDFOBn (CDCl_3_) 7

^1^H NMR spectrum of GDF_10_ (CDCl_3_) 8

^1^H NMR spectrum of GDF_14_ (CDCl_3_) 9

^1^H NMR spectrum of GDF_16_ (CDCl_3_) 10

FT-IR spectra of GDF_10_ 11

FT-IR spectra of GDF_14_ 12

FT-IR spectra of GDF_16_ 13

HRMS analysis of GDF_10_ 14

HRMS analysis of GDF_14_ 15

HRMS analysis of GDF_16_ 16

Epoxy Resins 17

^1^H NMR spectrum of GDF_10_EPO, GDF_14_EPO, GDF_16_EPO (CDCl_3_) 17

FT-IR spectrum of GDF_10_EPO, GDF_14_EPO, GDF_16_EPO 18

Epoxy Content of Epoxy Resins: Standard test method D1652 19

GPC analysis Raw data 20

Endocrine assays 22

Thermosets 23

TGA thermograms of the DA_10_ containing thermosets (10 °C/min under N_2_ flow) 23

TGA thermograms of the DIFFA containing thermosets (10 °C/min under N_2_ flow) 24

TGA thermograms of the IPDA containing thermosets (10 °C/min under N_2_ flow) 25

DSC curves of the DA_10_ containing thermosets 26

DSC curves of the DIFFA containing thermosets 27

DSC curves of the IPDA containing thermosets 28

DMA curves of the GDF_x_EPO:GTF / DIFFA thermosets 29

# Monomers

## Published synthetic pathways

**Synthesis of ethyl dihydroferulate:** Ferulic acid (250 g, 1.29 mol, 1 eq) was solubilized in ethanol (900 mL) and 2 mL of concentrated hydrochloric acid 37.5 w% (3.10-2 mol, 0.02 eq) was added. The mixture was stirred and heated at reflux for 48 h. The system was then cooled to 30 °C and put under inert atmosphere (nitrogen) before adding Pd/C 10 w% (5w%, 12.5 g). Mixture is stirred at room temperature for 48 h under hydrogen bubbling. The system was then filtered on Celite and the solvent was removed under vacuum, leading to the crude product which was then purified by silica gel flash chromatography eluted with cyclohexane/ethyl acetate (90/10) to afford the dihydroferulate as a white powder (97% yield).

**Synthesis of benzylated ethyl ferulate:** Ethyl ferulate (25 g, 0.1 mol, 1 eq), benzyl bromide (15 mL, 0.12 mol, 1.2 eq) and K_2_CO_3_ (27 g, 0.2 mol, 2 eq) were dissolved in dimethylformamide (DMF) (0.5 M) and heated to 85 °C. The reaction was monitored by TLC and let run until complete conversion of the starting material (3h). After cooling to room temperature (r.t.), the mixture was concentrated and filtered to remove K_2_CO_3_. The resulting phase was evaporated under reduced pressure and the crude product was purified by flash chromatography on silica gel using cyclohexane and ethyl acetate (90:10) as eluent, providing the desired product as a white powder (32 g, 92%).

**Synthesis of glycerol di-benzyl ferulate (GDFoBn):** Selective lipase-catalyzed transesterification was performed by using glycerol (5 g, 54.3 mmol, 1 eq), benzylated ethyl ferulate (42 g, 135.7 mmol, 2.5 eq) and CAL-B (10% w/w relative to the total weight of batch). The reaction mixture was heated to 75 °C, kept under reduced pressure and magnetically stirred for 3 days. It was then dissolved in acetone and filtered to remove CAL-B beads. The solvent was evaporated under vacuum and the crude product was purified by flash chromatography on silica gel eluted with cyclohexane/ethyl acetate to provide GDFoBn as a highly viscous oil (32 g, 93%).

**Synthesis of glycerol tri-ferulate (GTF): Glycerol:** (0.74 g, 8.01 mmol, 1 eq) and ethyl dihydroferulate (Figure 1, (b)) (8.08 g, 36.0 mmol, 4.5 eq) were melted and magnetically stirred at 75 °C before adding CAL-B (0.88 g, 10% by weight relative to the total weight of glycerol and ethyl dihydroferulate). The reaction mixture was kept under reduced pressure for 3 days. The reaction mixture was then dissolved in dichloromethane (100 mL) and filtered to remove CAL-B beads. The solvent was then evaporated under vacuum and the crude product was purified by flash chromatography on silica gel eluted with cyclohexane/ethyl acetate to provide GTF in 84% yield.

**Synthesis of lipophile glycerol di-ferulate (GDF_x_):** GDFoBn (figure 1 (d)) (20 g, 32.1 mmol, 1 eq) and fatty acid (lauric, palmitic or stearic, 1 eq) were dissolved in dichloromethane (DCM) (0.25 M) with a catalytic amount of DMAP, (1.1 g, 9.6 mmol, 0.3 eq). Subsequently, DIC (5.45 mL, 35.2 mmol, 1.1 eq) was added to the mixture and the reaction was magnetically stirred at r.t. overnight. The precipitate urea was removed via filtration and the filtrate concentrated under vacuum. The crude product was dissolved in THF and stirred under N_2_ flow at room temperature. After 10 min, palladium on activated charcoal (Pd/C, 10% w/w) was added and the solution was stirred under N_2_ for another 10 min, before being submitted to H_2_ flow to simultaneous reduce the C=C double bond and the benzyl protecting group (Bn). The solution was finally filtered using Celite® pads and evaporated under reduced pressure. Target bisphenol was purified by flash chromatography on silica gel eluted with cyclohexane/ethyl acetate. Structures were named **GDF_x,_** for **G**lycerol **D**i-**F**erulate, where the incrementation “x” indicates the alkyl chain length ( GDF_10_ 84% yield; GDF_14_ 81% yield; GDF_16_ 87% yield)..

**Synthesis of difurfurylamine (DIFFA)**

Furfurylamine (5 g, 51.5 mmol, 2.1 eq) was introduced in a two-necked roundbottom flask equipped with a reflux condenser and cooled down to 0 °C with an ice bath. A 18 _w_% aqueous solution of HCl (52 g, 0.25 mol, 5 eq.) was then added dropwise to furfurylamine. Once this addition was completed, the temperature was risen to 25 °C and the mixture was stirred for 15 min. Acetone (1.42 g, 24.5 mmol, 1.0 eq.) was then added to the mixture. The temperature was risen to 40 °C. In such a case, an additional aliquot of acetone (1 g, 0.4 eq) was added to the mixture. The reaction was stopped after 7 days and 2 additional aliquots of acetone were necessary to increase the conversion. The mixture was then cooled down to 25 °C and 150 mL of deionized water are added. The pH was adjusted to 10 with a 15_w_% aqueous solution of NaOH. The mixture was extracted thrice with ethyl acetate. The organic fractions were collected, washed with brine, dried over anhydrous magnesium sulfate and ethyl acetate was removed under vacuum to obtain a brown oil (74% yield).

## ^1^H NMR spectrum of benzylated ethyl ferulate (CDCl_3_)


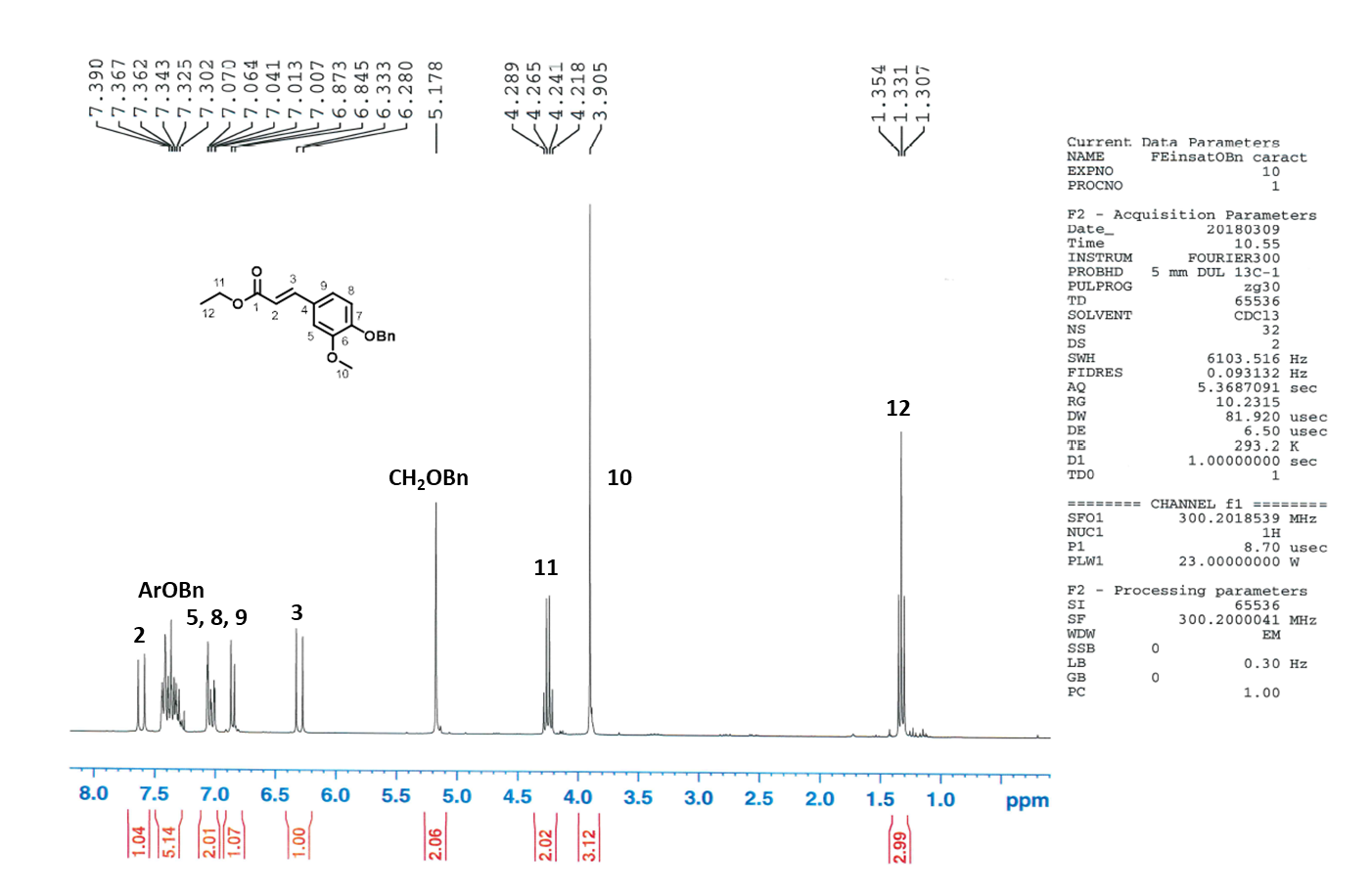


## ^1^H NMR spectrum of GDFOBn (CDCl_3_)


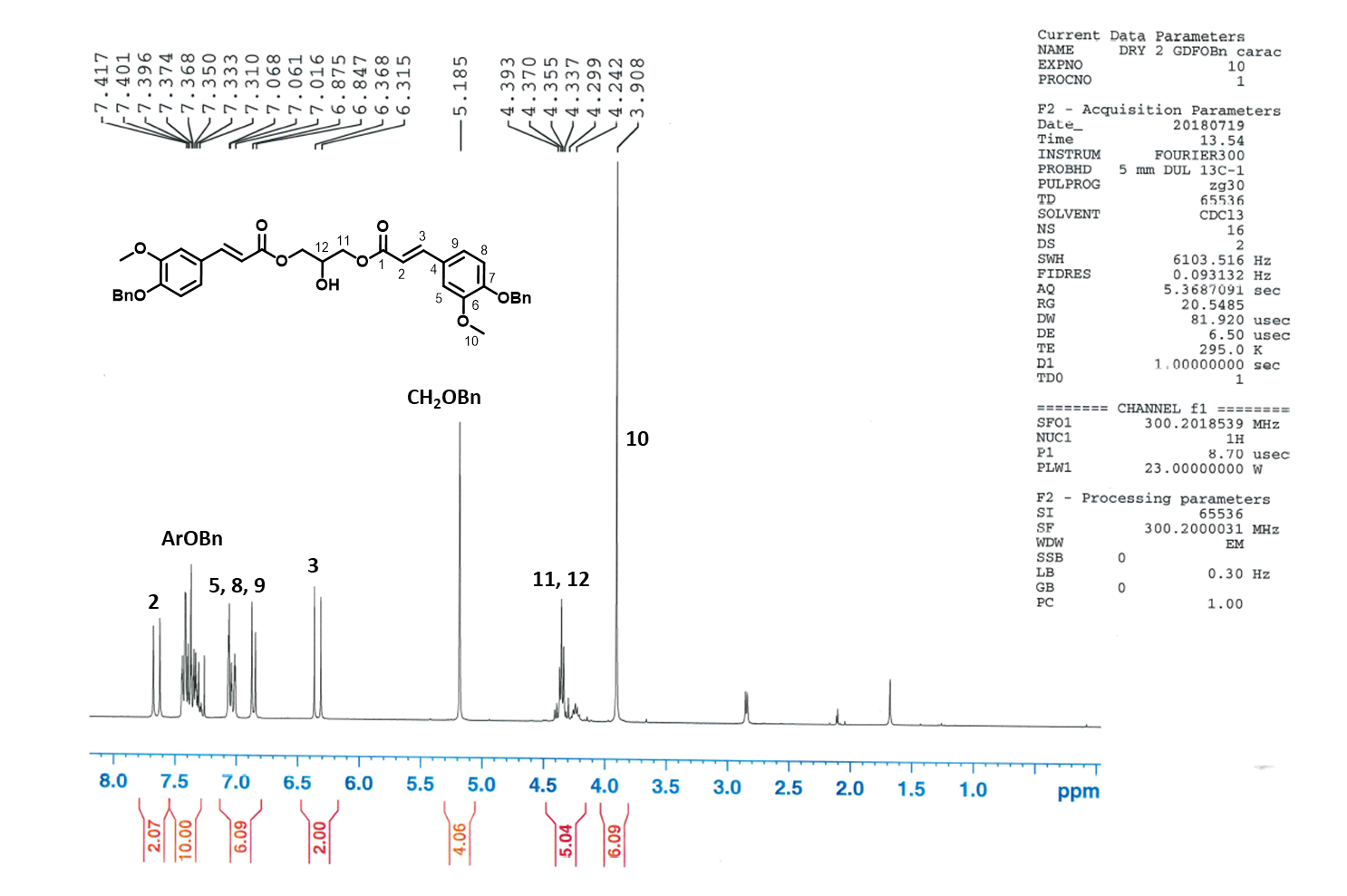


## ^1^H NMR spectrum of GDF_10_ (CDCl_3_)


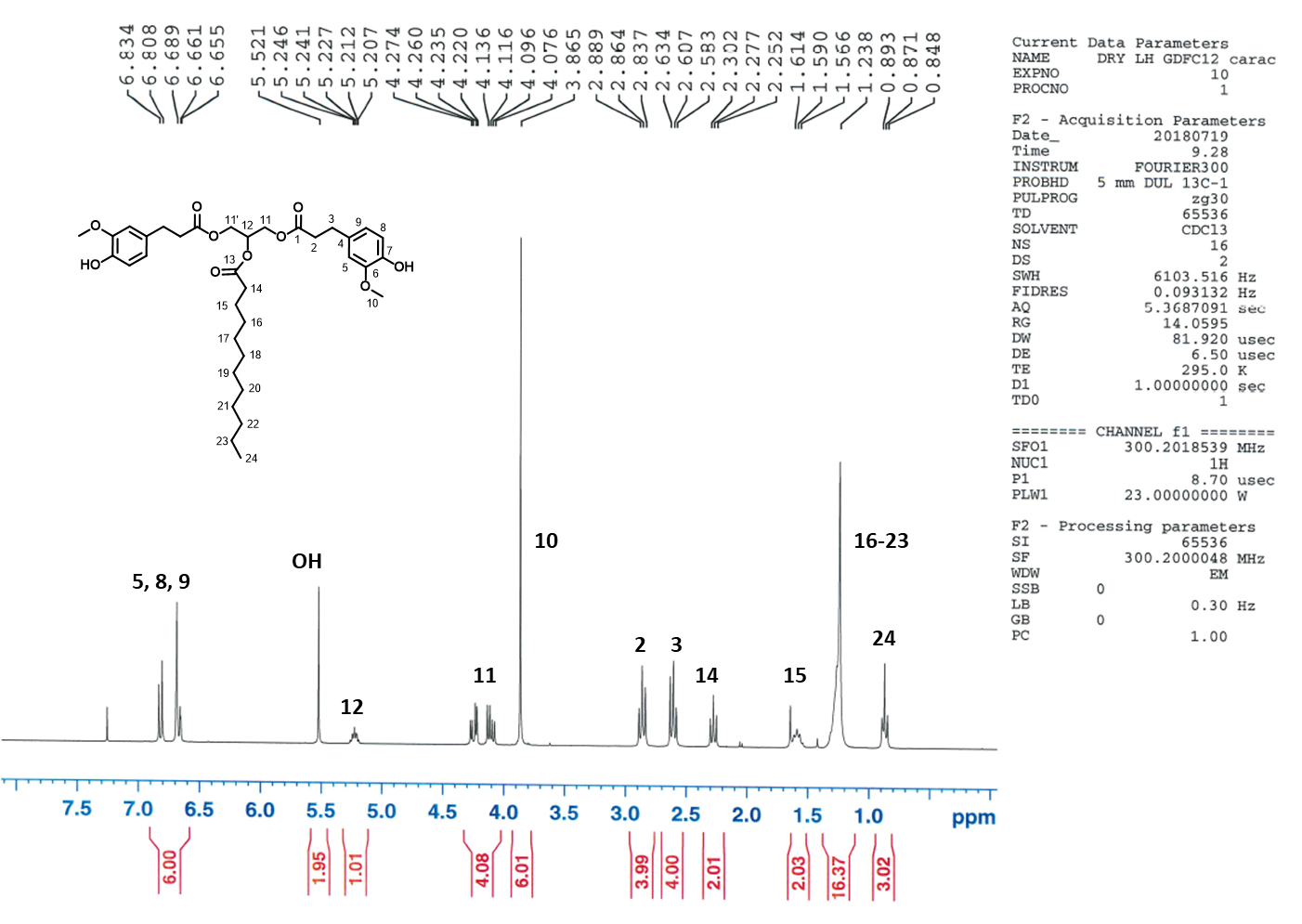


## ^1^H NMR spectrum of GDF_14_ (CDCl_3_)

_
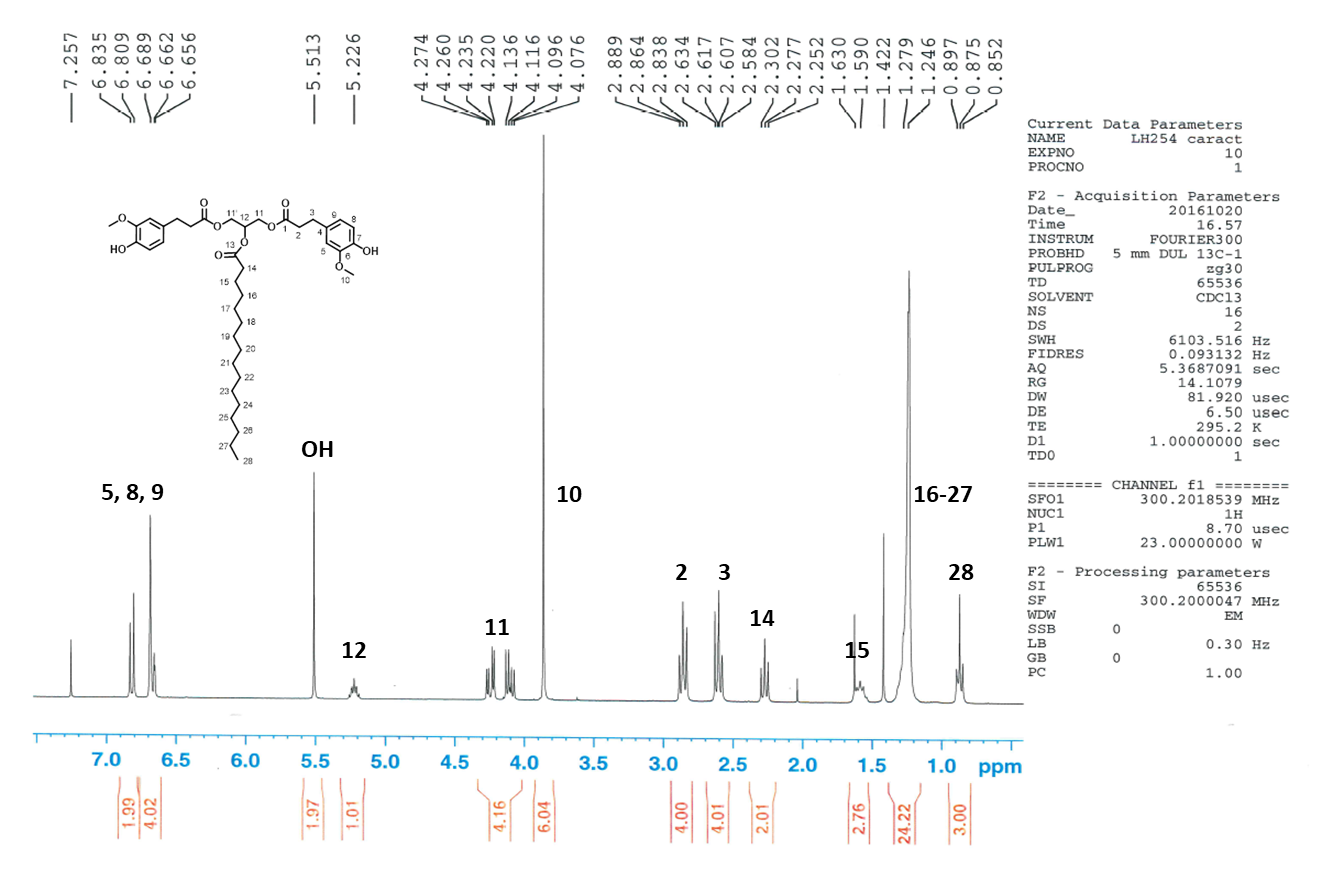
_

## ^1^H NMR spectrum of GDF_16_ (CDCl_3_)


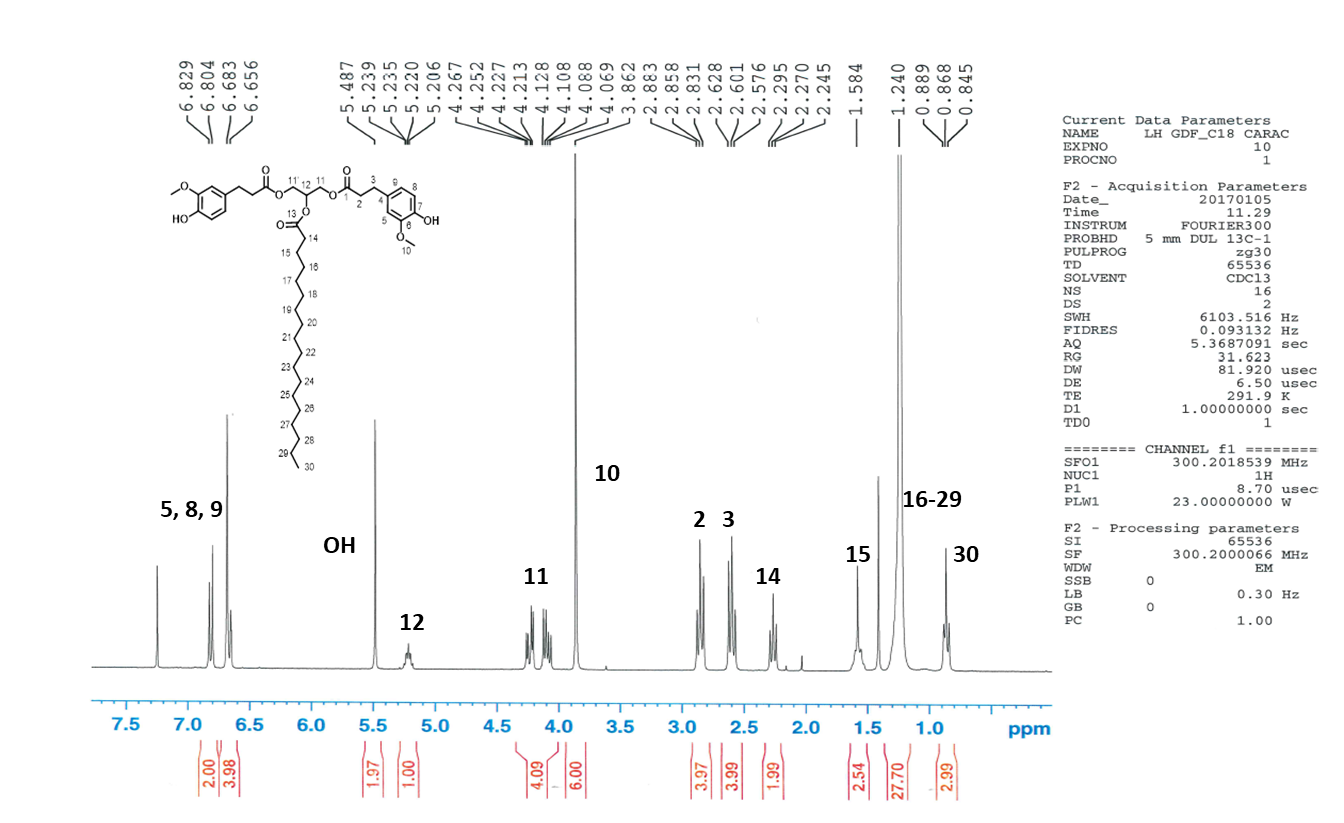


## FT-IR spectra of GDF_10_


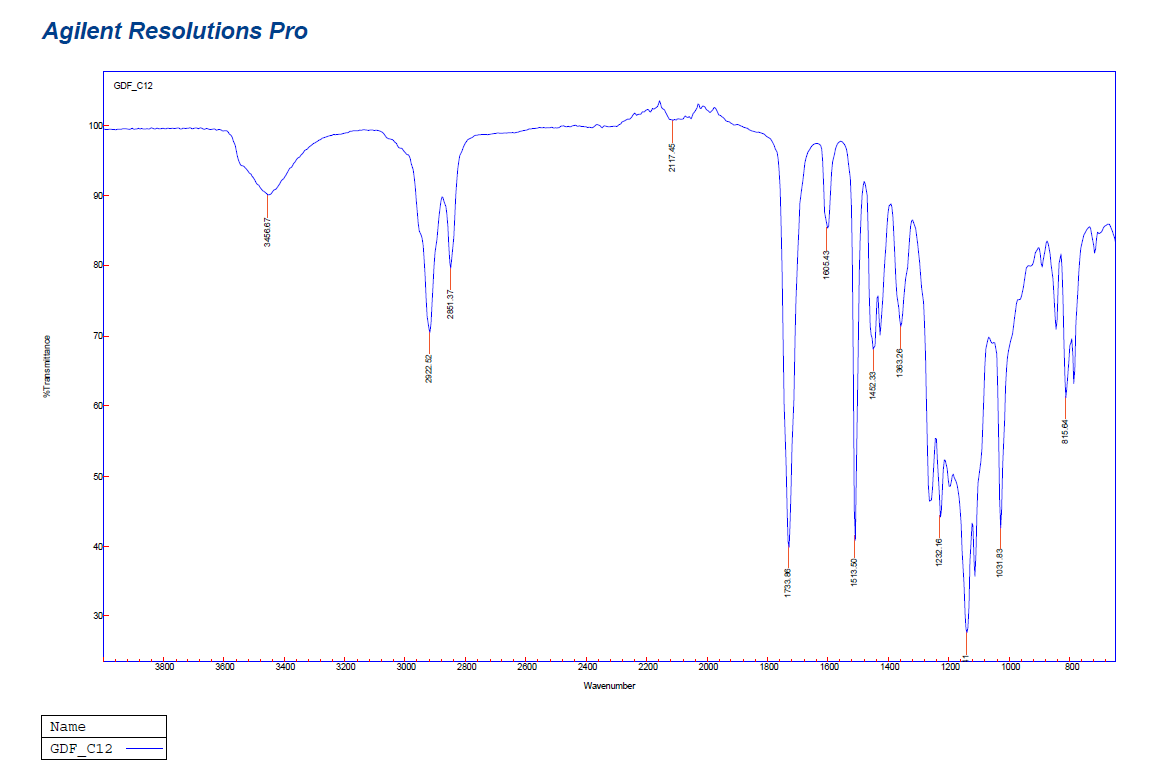


## FT-IR spectra of GDF_14_


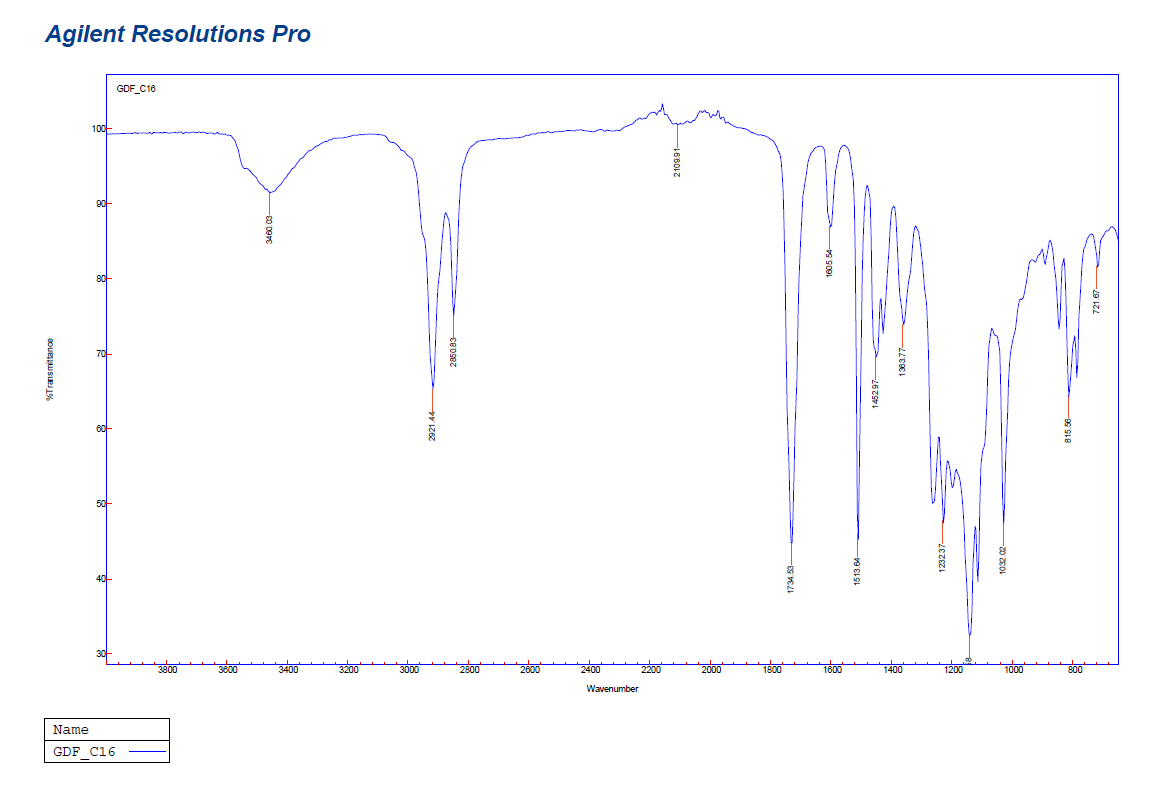


## FT-IR spectra of GDF_16_


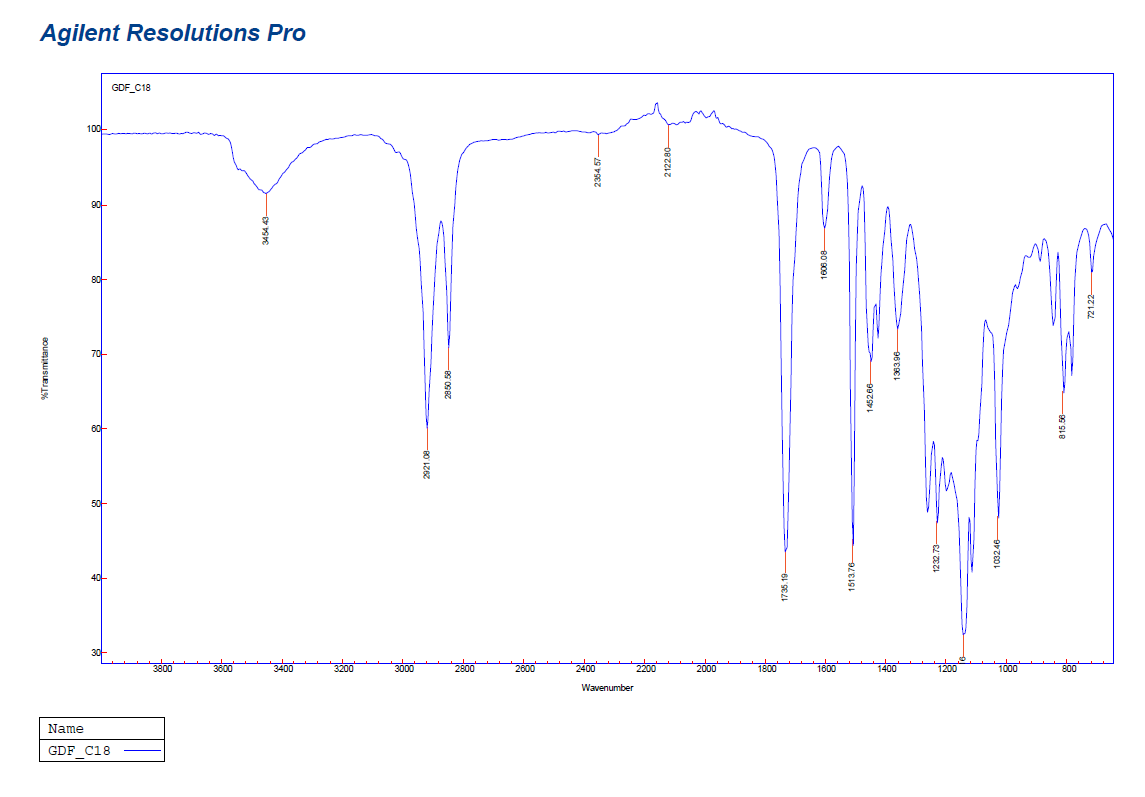


## HRMS analysis of GDF_10_


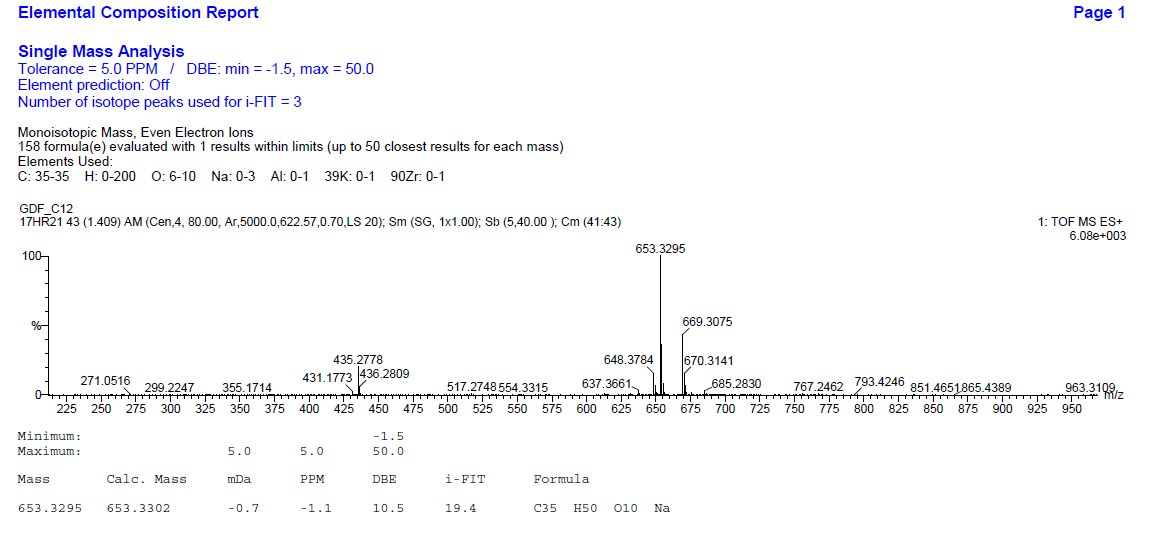


## HRMS analysis of GDF_14_


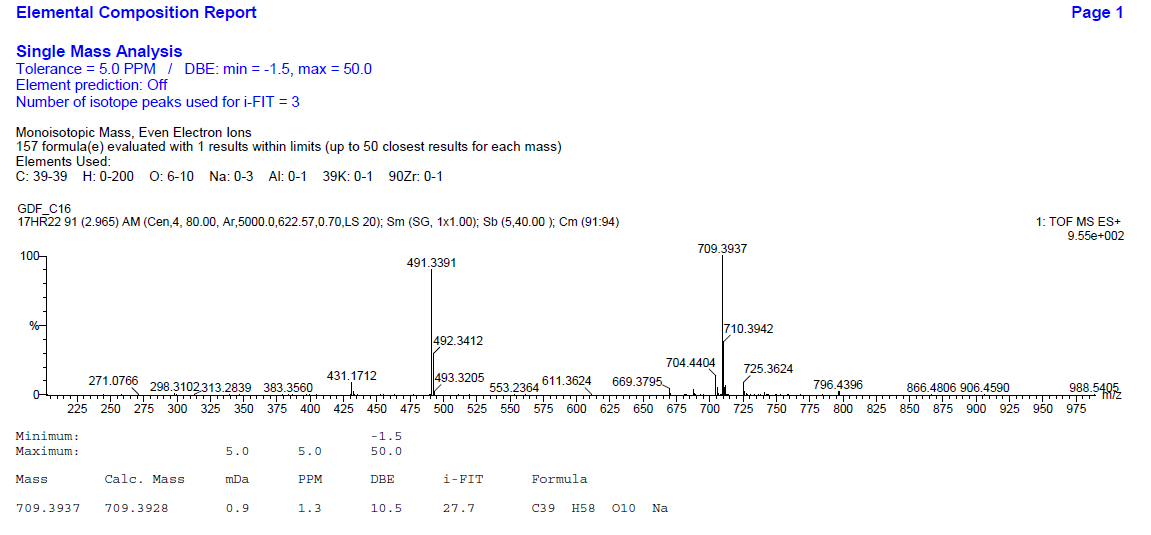


## HRMS analysis of GDF_16_


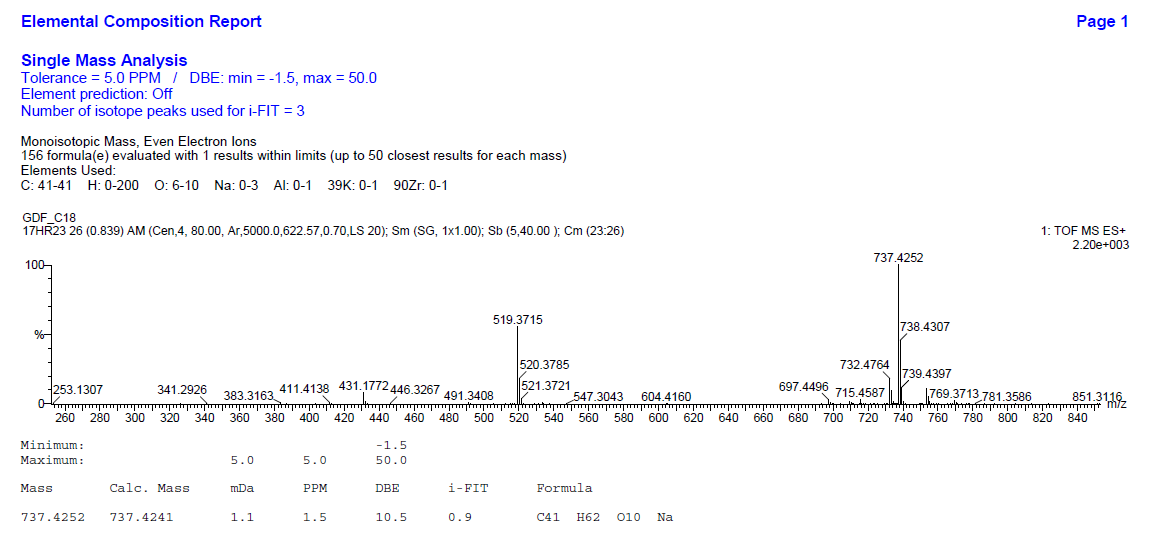


# Epoxy Resins

## ^1^H NMR spectrum of GDF_10_EPO, GDF_14_EPO, GDF_16_EPO (CDCl_3_)


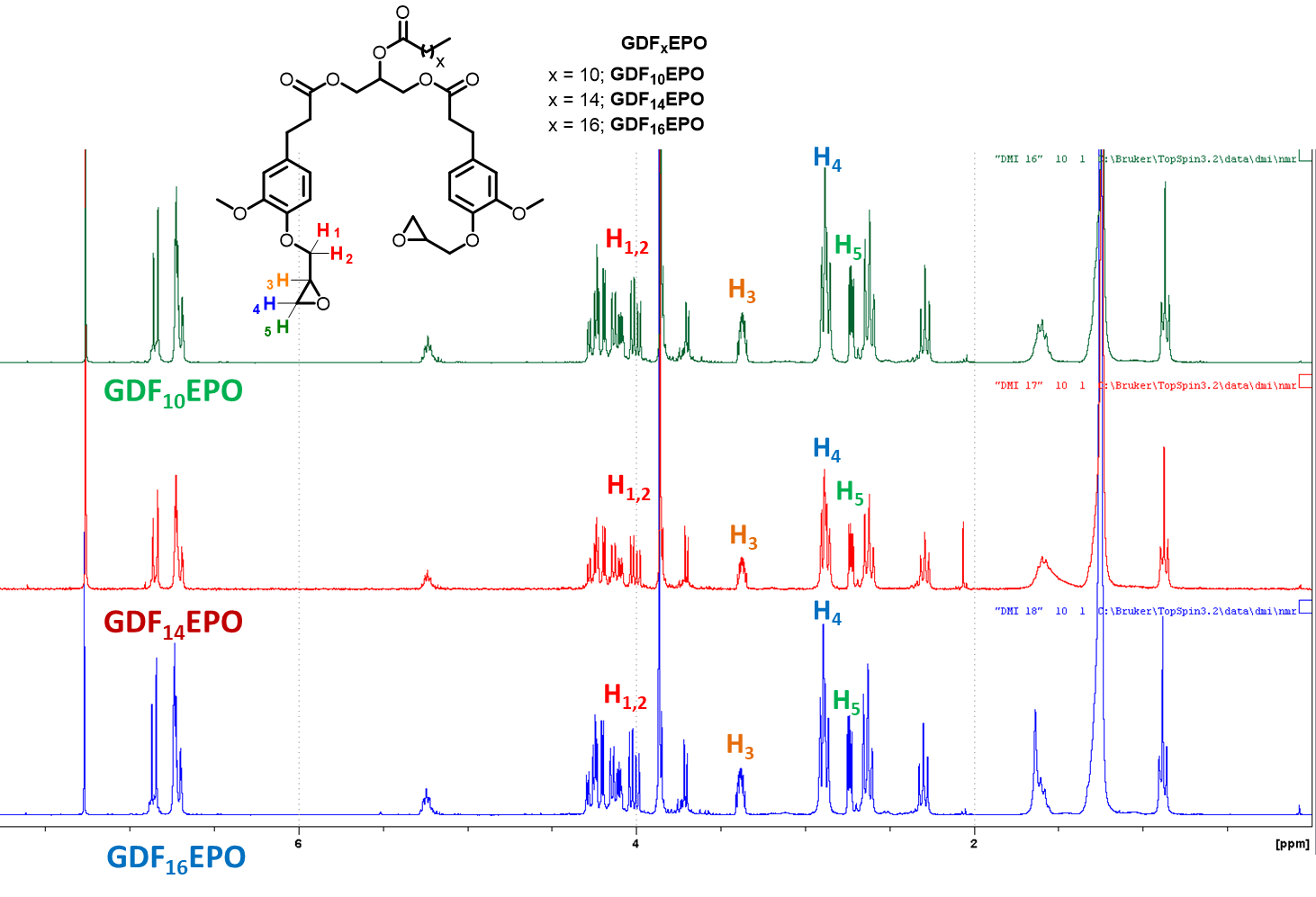


## FT-IR spectrum of GDF_10_EPO, GDF_14_EPO, GDF_16_EPO


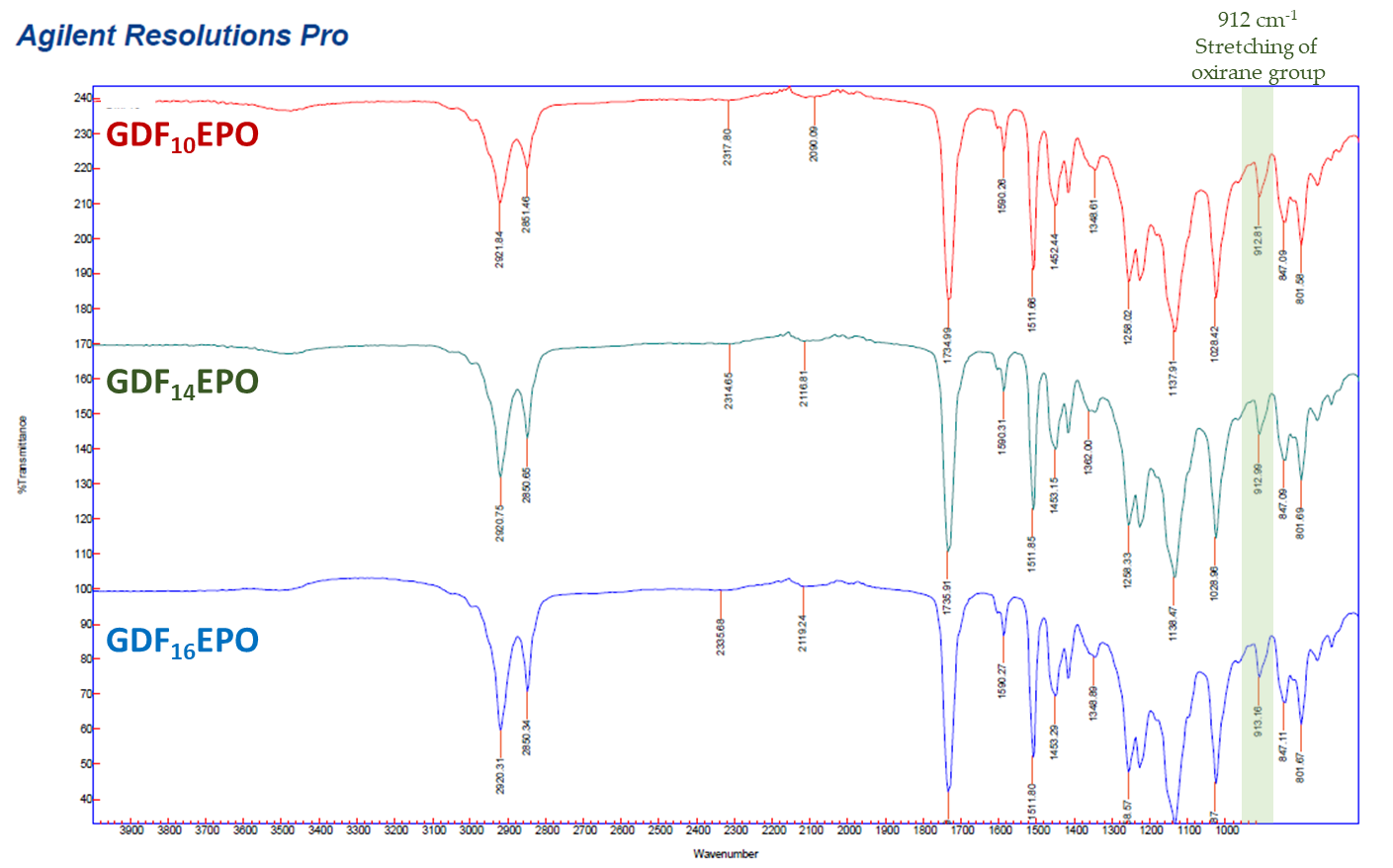


## Epoxy Content of Epoxy Resins: Standard test method D1652

ASTM international D1652

|  | **Weight (mg)** | **V_0_ (ml)** | **V_f_ (ml)** | **V_eq_ (mL)** | **E**_weight percent epoxide_ | **EEW** | **EEW** | **SD ±** |
| --- | --- | --- | --- | --- | --- | --- | --- | --- |
| GDF_10_EPO | 0.386 | 7 | 15.9 | 8.9 | 9.91 | 434 | 426 | 5.99 |
|  | 0.542 | 0 | 13 | 13 | 10.31 | 417 |  |  |
|  | 0.457 | 0 | 10.7 | 10.7 | 10.07 | 427 |  |  |
| GDF_14_EPO | 0.329 | 4.5 | 11.6 | 7.1 | 9.28 | 463 | 471 | 5.09 |
|  | 0.322 | 4.7 | 11.5 | 6.8 | 9.08 | 474 |  |  |
|  | 0.319 | 2.9 | 9.6 | 6.7 | 9.03 | 476 |  |  |
| GDF_16_EPO | 0.362 | 9.6 | 17.3 | 7.7 | 9.15 | 470 | 463 | 9.71 |
|  | 0.47 | 0 | 10 | 10 | 9.15 | 470 |  |  |
|  | 0.251 | 10 | 15.6 | 5.6 | 9.59 | 448 |  |  |
| GTF-EPO | 0.43 | 0 | 14.6 | 14.6 | 14.60 | 295 | 303 | 5.82 |
|  | 0.402 | 1 | 14.2 | 13.2 | 14.12 | 305 |  |  |
|  | 0.494 | 3.6 | 19.5 | 15.9 | 13.84 | 311 |  |  |

## GPC analysis Raw data

## Endocrine assays

(antagonist test)


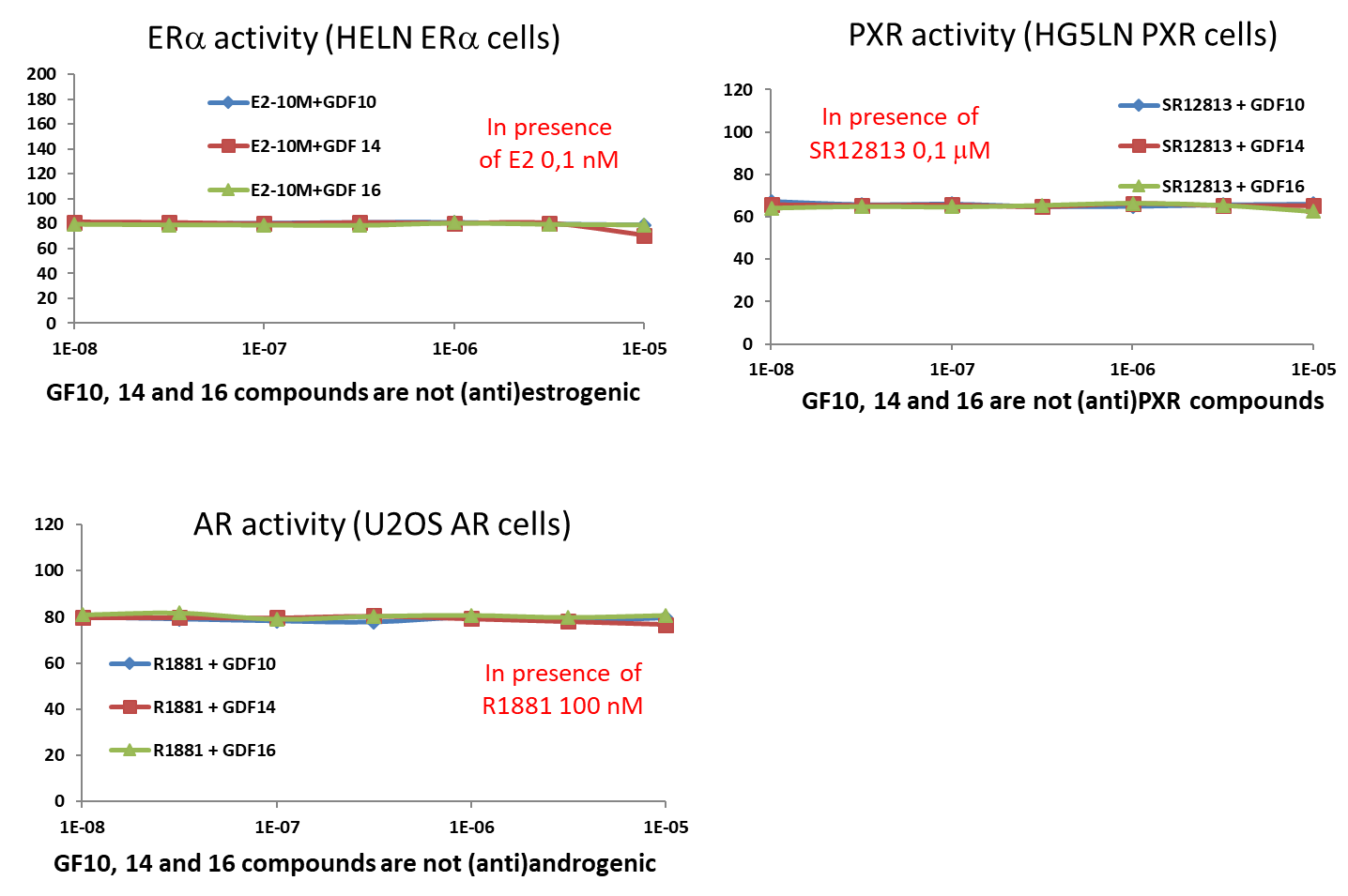


# Thermosets

## TGA thermograms of the DA_10_ containing thermosets (10 °C/min under N_2_ flow)


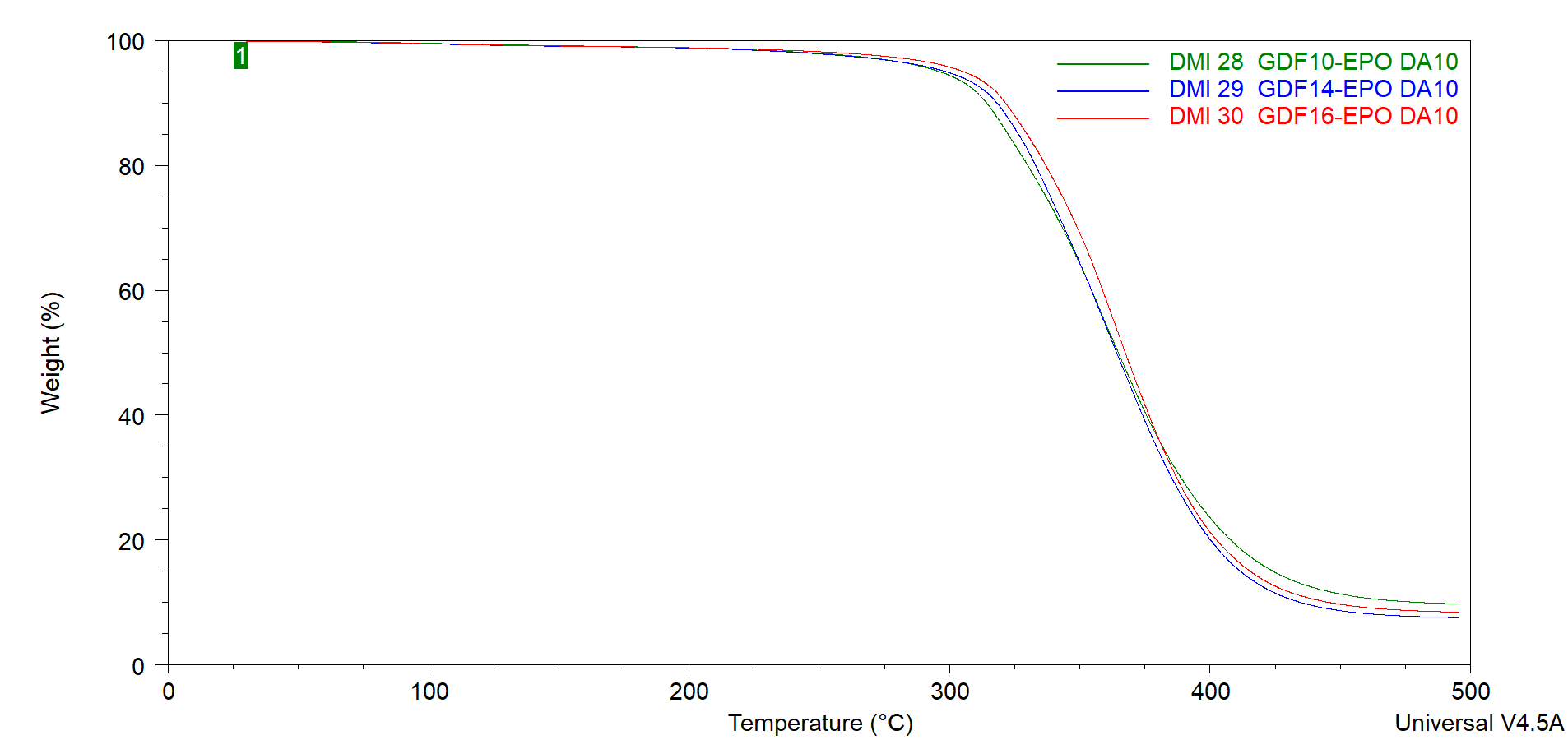


## TGA thermograms of the DIFFA containing thermosets (10 °C/min under N_2_ flow)


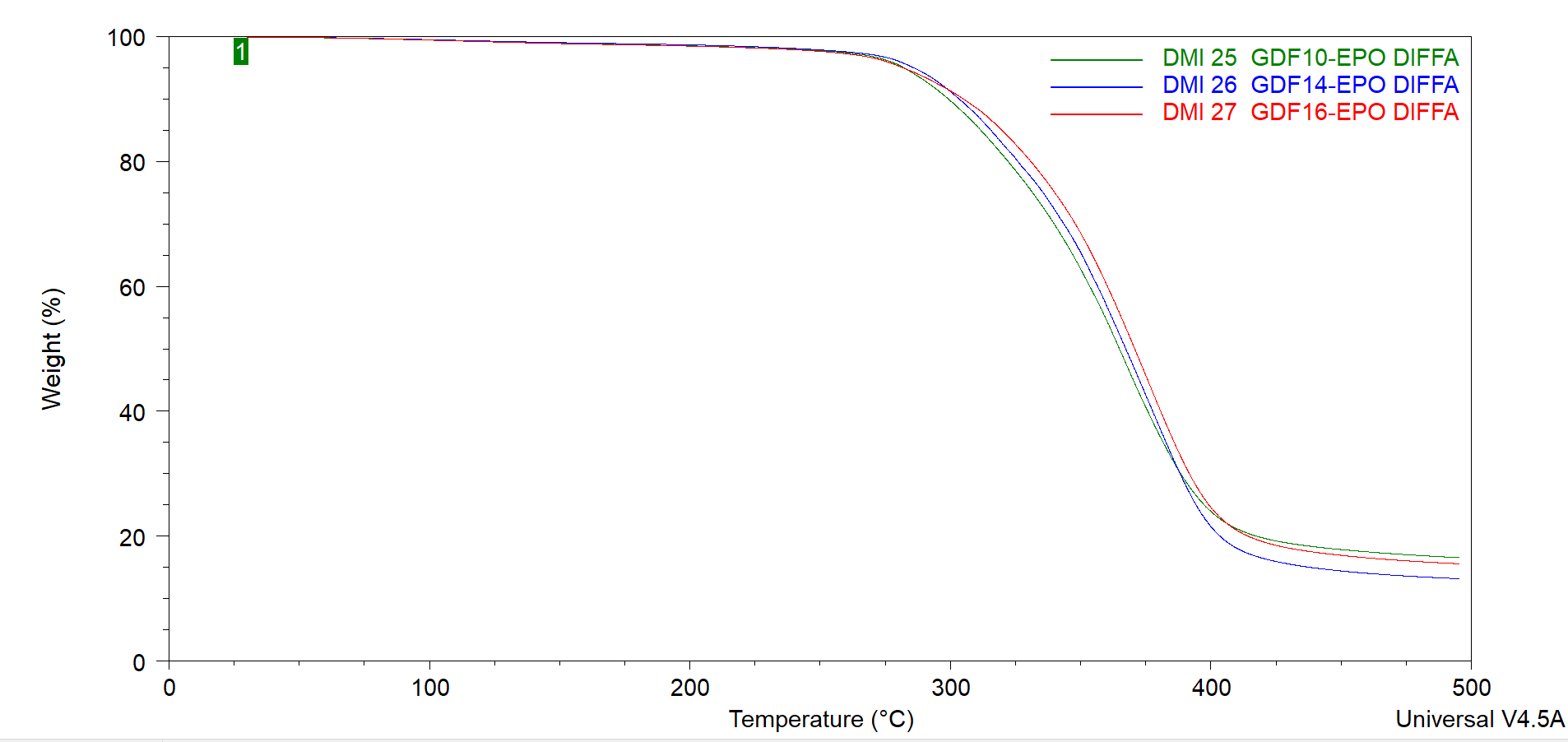


## TGA thermograms of the IPDA containing thermosets (10 °C/min under N_2_ flow)


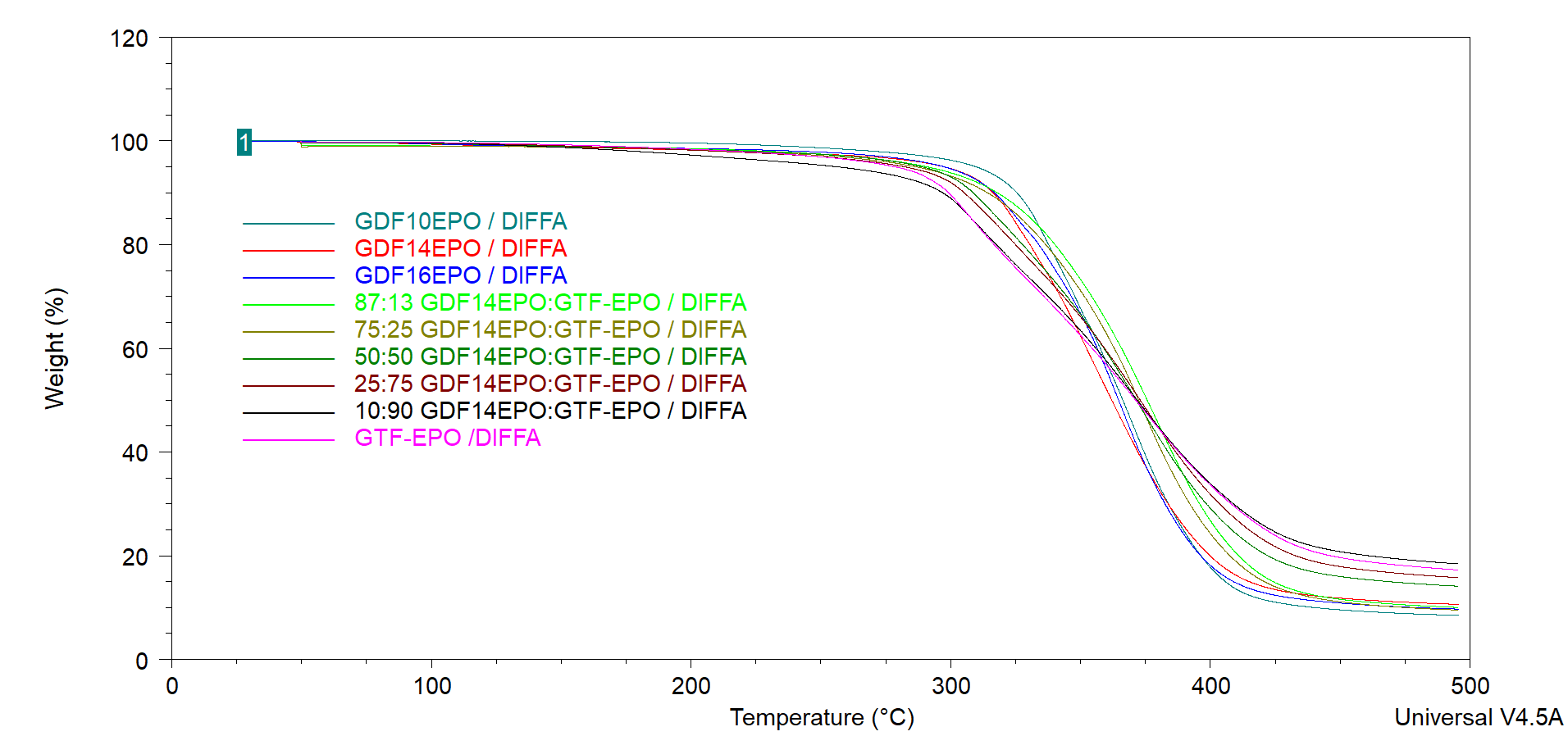


## DSC curves of the DA_10_ containing thermosets


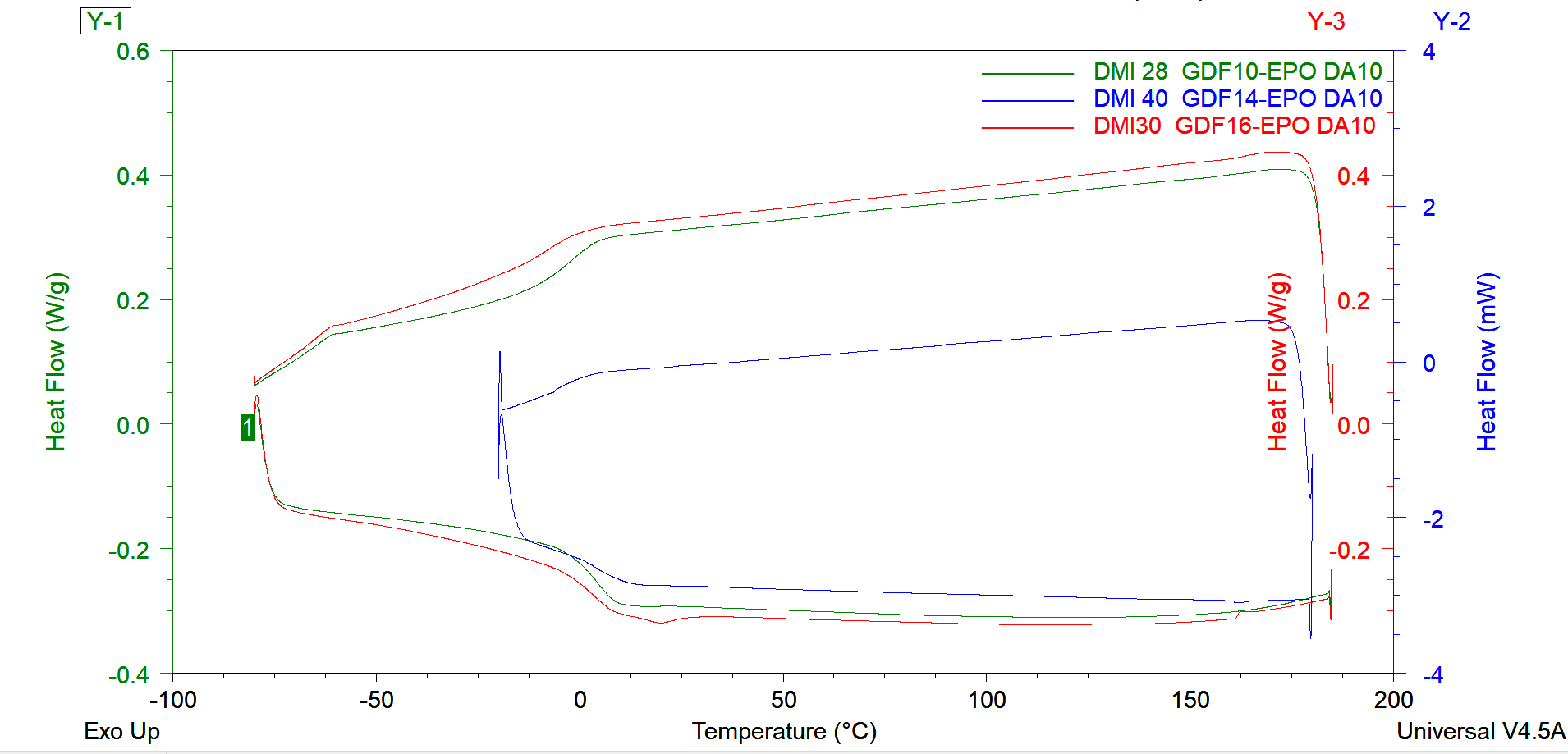


## DSC curves of the DIFFA containing thermosets


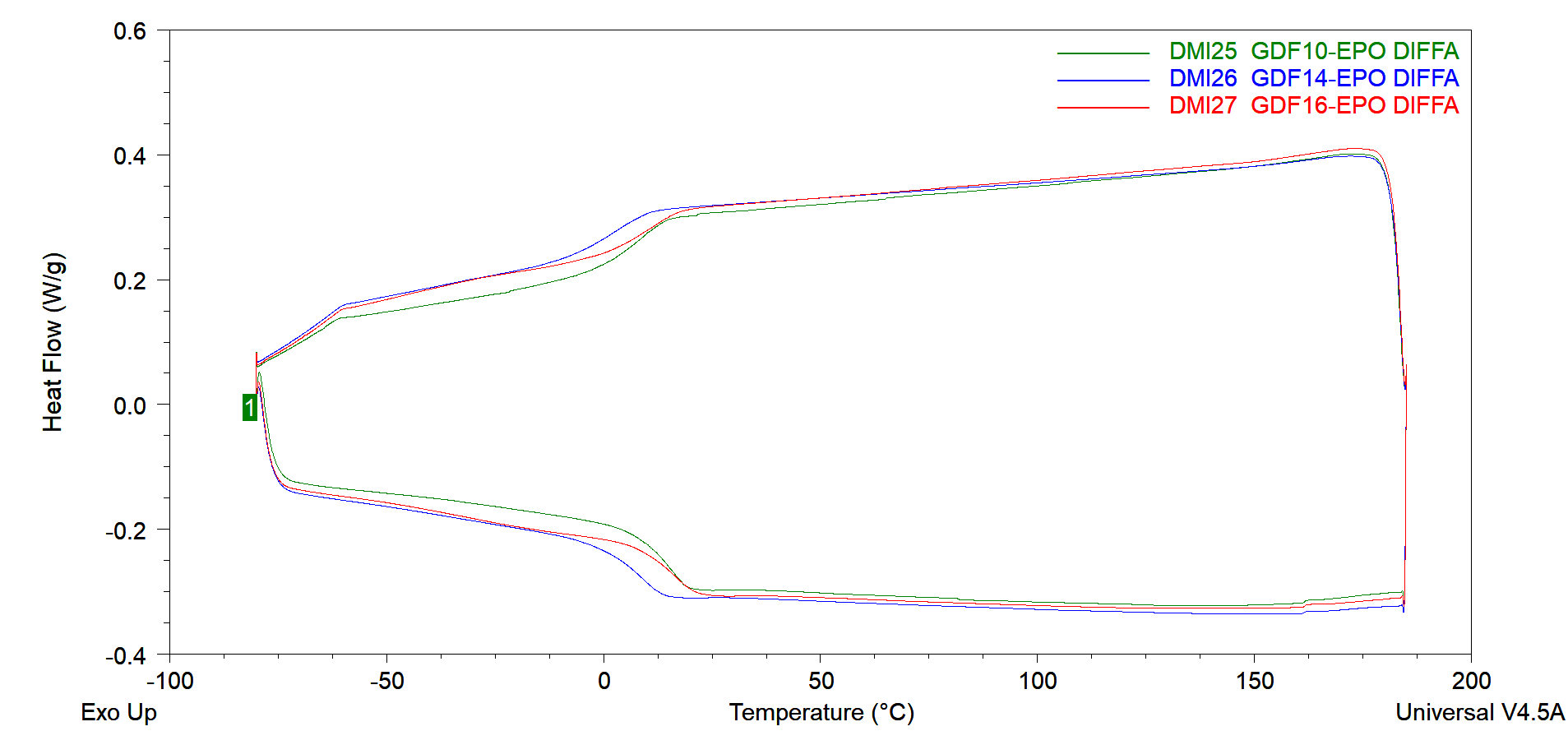


## DSC curves of the IPDA containing thermosets


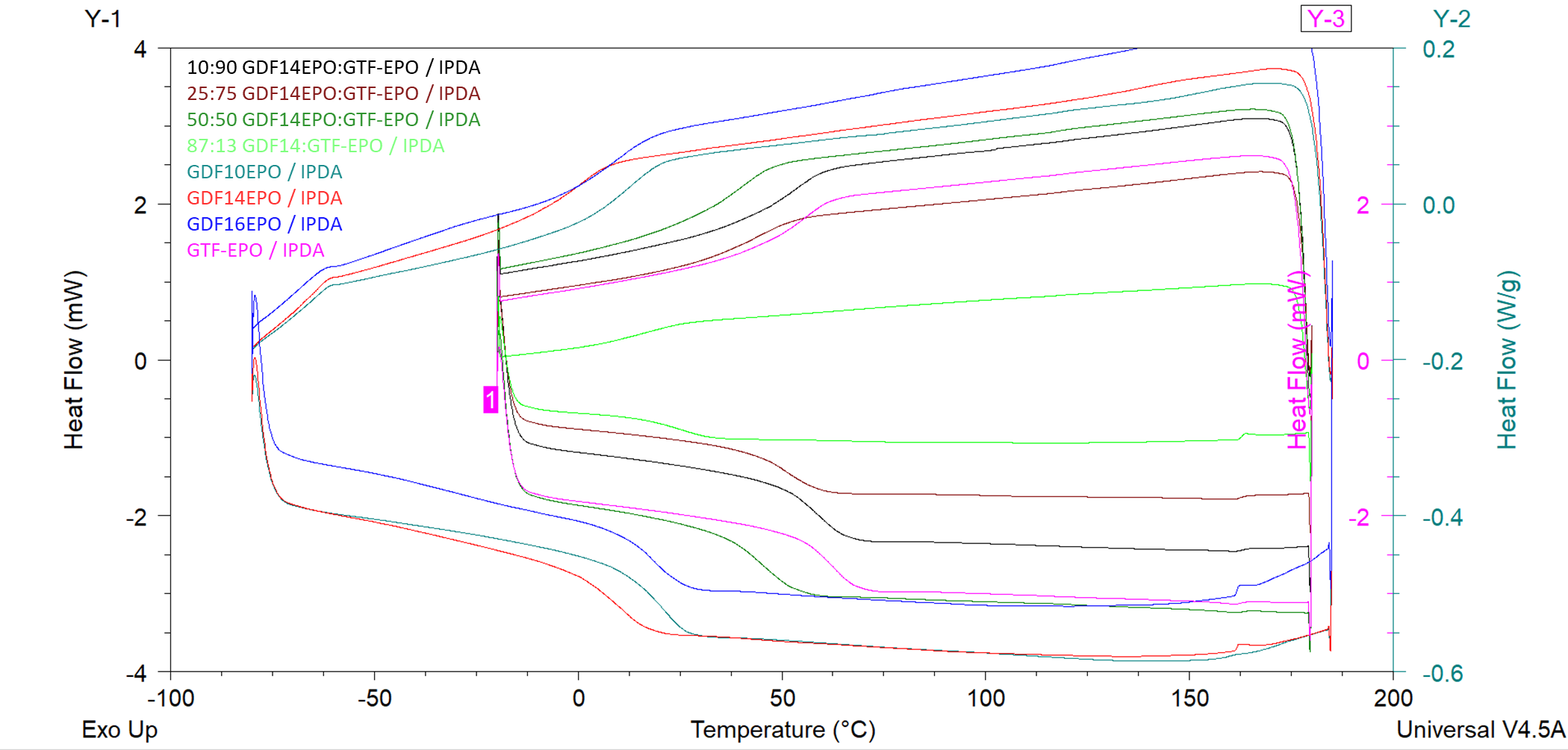


## DMA curves of the GDF_x_EPO:GTF / DIFFA thermosets


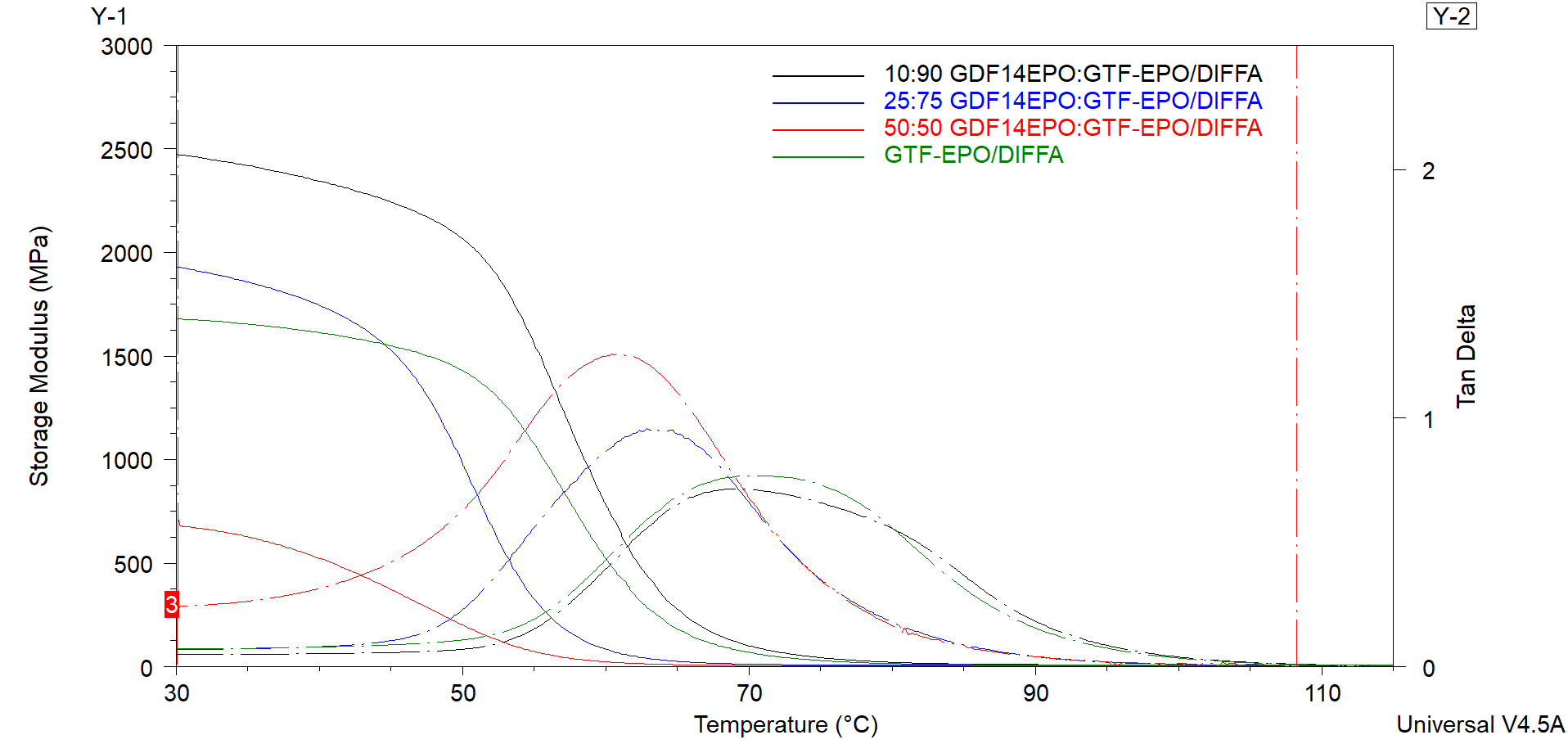

Supplement: Supplementary file 1 [file Data_Sheet_1.docx]
